# Large-scale Proteomic and Phosphoproteomic Analyses of Maize Seedling Leaves During De-etiolation

**DOI:** 10.1016/j.gpb.2020.12.004

**Published:** 2020-12-30

**Authors:** Zhi-Fang Gao, Zhuo Shen, Qing Chao, Zhen Yan, Xuan-Liang Ge, Tiancong Lu, Haiyan Zheng, Chun-Rong Qian, Bai-Chen Wang

**Affiliations:** 1 Key Laboratory of Photobiology, CAS, Institute of Botany, Chinese Academy of Sciences, Beijing 100093, China; 2 Vegetable Research Institute, Guangdong Academy of Agricultural Sciences, Guangdong Key Laboratory for New Technology Research of Vegetables, Guangzhou 510640, China; 3 Institute of Crop Cultivation and Farming, Heilongjiang Academy of Agricultural Sciences, Harbin 150086, China; 4 Beijing ProteinWorld Biotech, Beijing 100012, China; 5 Center for Advanced Biotechnology and Medicine, Biological Mass Spectrometry Facility, Rutgers University, Piscataway, NJ 08855, USA; 6 University of Chinese Academy of Sciences, Beijing 100049, China

**Keywords:** Maize seedling leaves, De-etiolation, Quantitative analysis, Proteome, Phosphoproteome

## Abstract

**De-etiolation** consists of a series of developmental and physiological changes that a plant undergoes in response to light. During this process light, an important environmental signal, triggers the inhibition of mesocotyl elongation and the production of photosynthetically active chloroplasts, and etiolated leaves transition from the “sink” stage to the “source” stage. De-etiolation has been extensively studied in maize (*Zea mays* L.). However, little is known about how this transition is regulated. In this study, we described a quantitative proteomic and phosphoproteomic atlas of the de-etiolation process in maize. We identified 16,420 proteins in proteome, among which 14,168 proteins were quantified. In addition, 8746 phosphorylation sites within 3110 proteins were identified. From the combined proteomic and phosphoproteomic data, we identified a total of 17,436 proteins. Only 7.0% (998/14,168) of proteins significantly changed in abundance during de-etiolation. In contrast, 26.6% of phosphorylated proteins exhibited significant changes in phosphorylation level; these included proteins involved in gene expression and homeostatic pathways and rate-limiting enzymes involved in photosynthetic light and carbon reactions. Based on phosphoproteomic analysis, 34.0% (1057/3110) of phosphorylated proteins identified in this study contained more than 2 phosphorylation sites, and 37 proteins contained more than 16 phosphorylation sites, indicating that multi-phosphorylation is ubiquitous during the de-etiolation process. Our results suggest that plants might preferentially regulate the level of posttranslational modifications (PTMs) rather than protein abundance for adapting to changing environments. The study of PTMs could thus better reveal the regulation of de-etiolation.

## Introduction

Proteotype is the proteomic state of a cell, and it reflects the integration of a cell’s genotype, developmental history, and environment [1]. Diversity of proteotype in a cell or tissue mainly comes from two forms: variants affecting the primary amino acid sequence and posttranslational modifications (PTMs) [2]. Although the genotype specifies the potential phenotype of an organism, proteins that implement cellular processes, and the interactions between these proteins and outside environment, dictate the actual phenotype. Therefore, to fully understand the biology of an organism and its constituent parts, the knowledge of proteotype or protein complement is required.

PTMs of the protein is an important component of the proteotype, which may affect protein functions, such as protein phosphorylation. Generally speaking, a newly synthesized protein may not have a biological function until it is modified [3]. PTMs provide a more precise and elegant mechanism to control cellular function than regulation of gene expression [4]. For instance, PINFORMED1 (PIN1) shows a tissue-specific difference in phosphorylation in the maize leaf that correlates with changes in polarized localization of PIN1 in epidermal cells during development [5]. In PTM databases, more than 300 different types of PTMs have been described and the number is still increasing [6].

Protein phosphorylation is an important type of PTMs, which has been extensively studied since it was first reported in 1926 [7]. According to published data, protein phosphorylation is one of the most abundant PTMs in all biological species, representing 53.5% of all PTMs [8]. The conversion between phosphorylation and dephosphorylation of specific sites can alter the molecular conformation of the protein, potentially affecting enzyme activity, substrate specificity, structural stability, or intracellular localization, and thus the regulation of biological processes [8], [9].

Many proteins contain multiple phosphorylation sites. On one hand, different phosphorylation sites can regulate different functions of the target protein. For example, phosphorylation of proteins at different sites activates or inhibits their activities [10]. On the other hand, a combination of multiple phosphorylation sites that have similar functions in the same protein may amplify the effect of phosphorylation. Moreover, phosphorylation of multiple sites on the same protein can function as a molecular switch that allows biological crosstalk between different redundant and alternative pathways [11]. Phosphoproteome analysis, which includes identification of phosphorylated proteins, exact mapping of phosphorylation sites, quantification of phosphorylation, and identification of the associated biological functions, is an effective approach for analyzing these biological regulatory networks at a global level [9], [12].

Seedling de-etiolation is a complex but precisely regulated process. During subterranean growth, dark-grown or etiolated seedlings have fast-growing hypocotyls (dicots) or mesocotyls (monocots) that allow them to rapidly reach the light, together with a protective apical hook and appressed cotyledons (dicots) or a protective coleoptile (monocots) with undeveloped chloroplasts. At the soil surface, incident light represses hypocotyl or mesocotyl elongation and stimulates cotyledon separation (dicots) or leaf expansion (monocots), congruent with the development of functional chloroplasts, thus enabling light capture for photosynthesis [13], [14]. Several key regulators of the de-etiolation process have been identified, including constitutive photomorphogenic 1 (COP1), elongated hypocotyl 5 (HY5), and phytochrome-interacting factors (PIFs), which play essential roles in regulating the massive reprogramming of the plant transcriptome during de-etiolation [15], [16], [17]. Moreover, phosphorylation modification plays an essential role in the regulation of these key regulators. For example, phytochromes (PHYs) are unphosphorylated and located in the cytosol in the dark. After illumination with light, they are converted to active P_fr_ forms and phosphorylated, and then rapidly localize to the nucleus where they phosphorylate downstream proteins, such as PIFs. Phosphorylated PIFs are targeted to the proteasome and degraded, resulting in the promotion of photomorphogenesis [17], [18].

Previous studies have indicated that a significant portion of the genome, at least 20% in both *Arabidopsis* and rice, is differentially expressed between seedlings that are undergoing photomorphogenesis and skotomorphogenesis [19], [20]. However, it has become clear that mRNA levels are poorly correlated with protein abundance [1], [21]. To bridge this gap, proteomics studies have been performed on *Arabidopsis* [22], rice [23], [24], and maize [25] seedlings undergoing de-etiolation. However, due to the limited ability to identify and quantify protein phosphorylation using the proteomic methods available at the time these studies were performed, only several dozen proteins were found to have differences in protein phosphorylation levels. Therefore, a much deeper proteomic survey is needed to reveal the mechanism by which phosphorylation regulates seedling de-etiolation.

Besides being the world’s largest crop in terms of production, maize is also an important model plant for basic research, especially as a C4 model plant for photosynthesis research. The completion of the B73 maize genome [26] has facilitated the use of large-scale transcriptome and proteome data to reveal the mechanisms underlying various maize developmental and physiological processes. For example, researchers have created large data resources for C4 photosynthesis research, including complementary RNA-seq [27], proteomics [28], [29], and phosphoproteomics [30], [31] data for a developmental gradient of the maize leaf. In these studies, the mRNA and protein contents at successive stages of photosynthetic development were analyzed. However, our understanding of how the proteome changes during a given developmental process of maize is still incomplete. In the present study, we performed 3D-HPLC-MS/MS and 2D-HPLC-MS/MS to obtain deep proteomic information for maize seedlings undergoing de-etiolation, and used two methods, immobilized metal ion affinity chromatography (IMAC) and TiO_2_, to enrich phosphorylated peptides to obtain deep phosphoproteomic information. Our results provide abundant data for better understanding the regulation of de-etiolation in maize.

## Results and discussion

### Strategy for quantitative analyses of the maize leaf proteome and phosphoproteome

**Figure 1:**
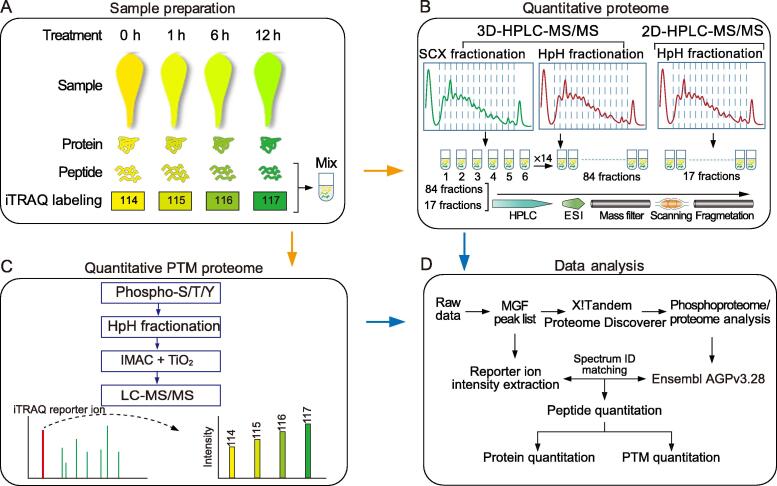
**Experimental workflow for the proteome and phosphoproteome analyses of de-etiolated maize seedling leaves** **A.** Sample preparation. Etiolated maize seedlings (B73 inbred) were illuminated with white light, and the first leaves were harvested after 0 h, 1 h, 6 h, and 12 h. Total protein was extracted from each sample. After trypsin digestion, peptides from each sample were labeled with one of four iTRAQ tags, and then peptides from all samples were combined. **B.** The analysis of iTRAQ-labeled peptides with 3D-HPLC-MS/MS and 2D-HPLC-MS/MS. In 3D-HPLC-MS/MS analysis, iTRAQ-labeled peptides were first separated into six fractions by SCX chromatography, and each primary fraction was then separated into 14 secondary fractions using HpH RPC. In 2D-HPLC-MS/MS analysis, iTRAQ-labeled peptides were separated into 17 fractions by HpH RPC before being subjected to HPLC-MS/MS. **C.** Enrichment and purification of phosphorylated peptides. **D.** Spectra were searched against annotated databases. SCX, strong cation exchange; HpH RPC, high pH reversed-phase chromatography; ESI, electron spray ionization; PTM, posttranslational modification; Phospho-S/T/Y, peptides containing phosphorylation sites on Ser/Thr/Tyr; IMAC, immobilized metal ion affinity chromatography; TiO_2_, Titanium dioxide; MGF, Mascot Generic Format.

**Figure 2:**
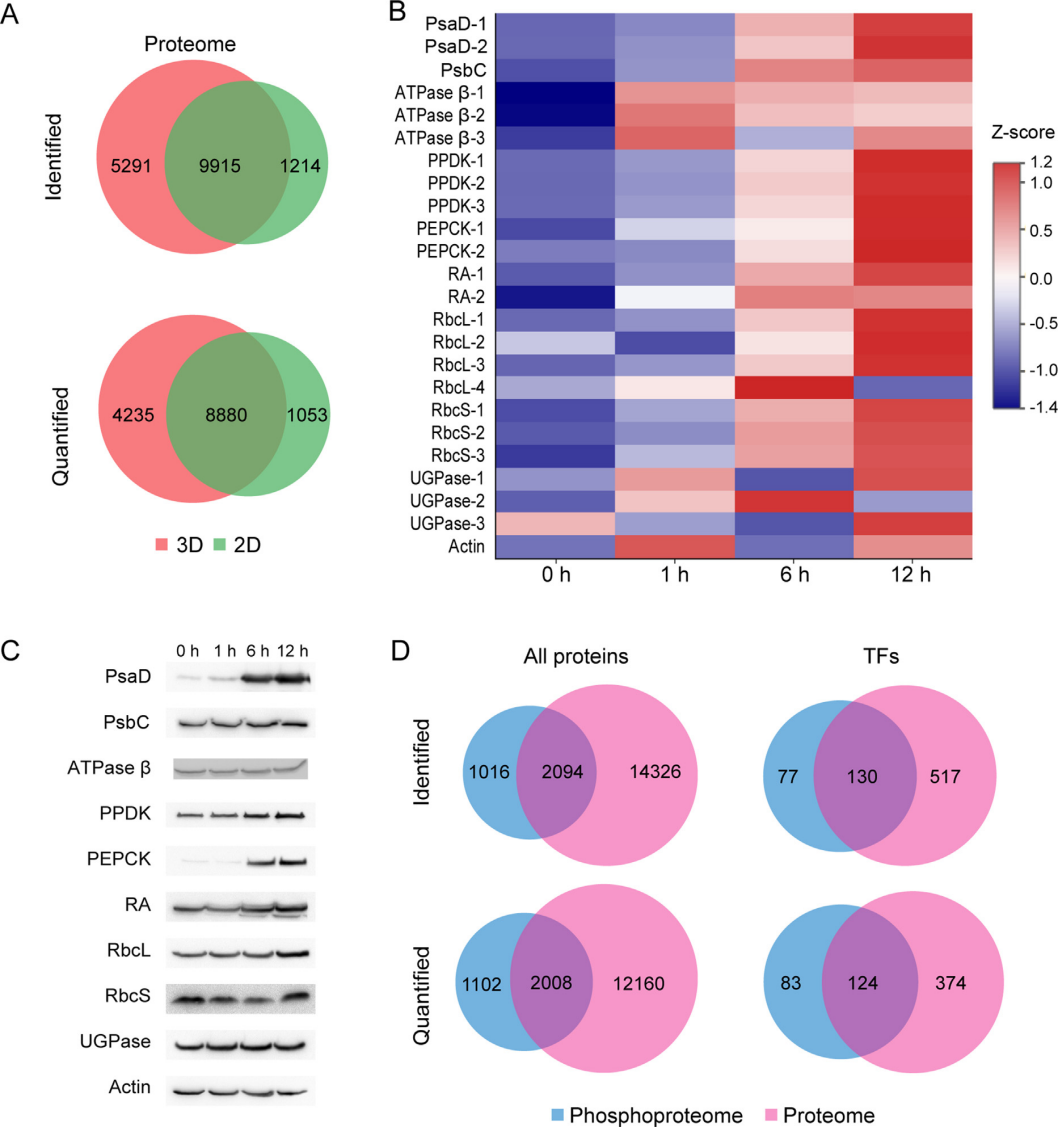
**Overview of the number of proteins and phosphorylatedproteins identified in de-etiolated maize seedling leaves** **A.** Venn diagram showing the number of overlapping proteins identified (top) and quantified (bottom) in de-etiolated maize seedling leaves using 2D- and 3D-HPLC-MS/MS. **B.** Heat map illustrating the dynamic changes in expression of 10 kinds of quantified proteins. The relative protein abundance (the ratio to 0 h) was normalized by Z-score standardization. **C.** Western blot analysis of the 10 kinds of quantified proteins shown in (B). **D.** Venn diagrams showing the number of overlapping proteins (left) and TFs (right) identified (top) and quantified (bottom) in the proteomic and phosphoproteomic analyses of de-etiolated maize seedling leaves. FC, fold change;  PsaD, D subunit of the PSI complex; PsbC, C subunit of the PSII complex; ATPase β, the β subunit of ATPase; PPDK, pyruvate orthophosphate dikinase; PEPCK, phosphoenolpyruvate carboxykinase; RA, Rubisco activase; RbcL, large subunit of Rubisco; RbcS, small subunit of Rubisco; UGPase, UDP-glucose pyrophosphorylase; TF, transcription factor.

To perform a deep analysis of the maize proteome and phosphoproteome during de-etiolation, the first leaves of 7-day-old etiolated maize seedlings (B73 inbred) were illuminated with white light and harvested at 0 h, 1 h, 6 h, and 12 h for mass spectrometry analysis (Figure 1). We chose these samples for analysis because in our previous studies [25], [31], [32] we found that the first leaves of 7-day-old etiolated maize seedlings showed no significant changes in growth compared with light-grown seedlings when subjected to illumination for 12 h, and few proteins involved in development and growth were found to be differently expressed when comparing the proteomes of etiolated and normal leaves. Moreover, the etiolated leaves turned green after 12 h of illumination, which indicated that processes related to greening in leaves were completed. Total protein was extracted from each sample followed by trypsin digestion. The peptides obtained from each sample were labeled with one of four iTRAQ tags, and then peptides from all samples were combined (Figure 1A). To obtain deep proteomic information, the iTRAQ-labeled peptides were analyzed with 3D-HPLC-MS/MS. Specifically, the labeled peptides were first separated into six primary fractions by strong cation exchange (SCX) chromatography, and each primary fraction was then separated into 14 secondary fractions using high pH reversed-phase chromatography (HpH RPC) (Figure 1B). Finally, the resulting 84 fractions were analyzed with HPLC-MS/MS. The spectra were analyzed and filtered at 1% FDR. In total, 15,206 proteins were identified. Of these proteins, 13,115 were quantified with at least one uniquely mapped peptide and at least two quantifiable spectra (Figure 2A; Table S1). To validate this result, the maize leaf proteome was reanalyzed in two independent experiments using 2D-HPLC-MS/MS, in which iTRAQ-labeled peptides were separated into 17 fractions using HpH RPC and then analyzed with HPLC-MS/MS (Figure 1B). Using the same criteria mentioned above, 11,129 proteins were identified and 9933 proteins were quantified using 2D-HPLC-MS/MS (Figure 2A; Table S1). The Pearson correlation coefficients determined by comparing the protein abundances from the 3D proteome analysis with those from replicates 1 and 2 of the 2D proteome analysis were 0.77 and 0.75, respectively, demonstrating that there was a good correlation between the two methods (Figure S1). A total of 9915 proteins were identified with both 2D- and 3D-HPLC-MS/MS, and of these proteins, 8880 were quantified (Figure 2A). Moreover, the dynamic changes of 10 kinds of quantified proteins determined from HPLC-MS/MS data were confirmed by Western blot analysis (Figure 2B and C; Table S1). Taken together, using the two approaches, we identified a total of 16,420 proteins encoded by 15,653 genes, and of these proteins, 14,168 encoded by 13,612 genes were quantified.

Characterization of myriad PTMs is another key aspect of proteome profiling. Here, we exhaustively studied one important PTM event, Ser/Thr/Tyr phosphorylation (pS/pT/pY), in de-etiolated maize seedling leaves. Phosphorylated peptides were enriched using IMAC and TiO_2_ in parallel, and these peptides were then combined for LC-MS/MS analysis (Figure 1C). The spectra were searched against the *Zea mays* database using MASCOT or MSAmanda in the Proteome Discoverer environment. By using stringent cutoff criteria (see Materials and methods), phosphorylation on 8746 S/T/Y residues (sites) representing 3110 proteins was quantified (Figure 2D; Table S2). We quantified 1102 phosphorylated proteins that did not overlap with the 14,168 quantified proteins, suggesting that most are low abundant proteins that could not be identified without enrichment (Figure 2D; Table S3). To prevent possible biases due to variation in protein expression, the relative intensities of the phosphorylated peptides were normalized against changes in protein abundance [33]. Finally, 2008 proteins with normalized phosphorylation levels (NPLs) were used for further analysis of phosphorylation dynamics.

Integrating the proteome and phosphoproteome results, we identified a total of 17,436 proteins encoded by 15,970 genes, including 724 transcription factors (TFs) encoded by 663 genes (Figure 2D; Table S4).

### Dynamic reprogramming of the maize leaf proteome

**Figure 3:**
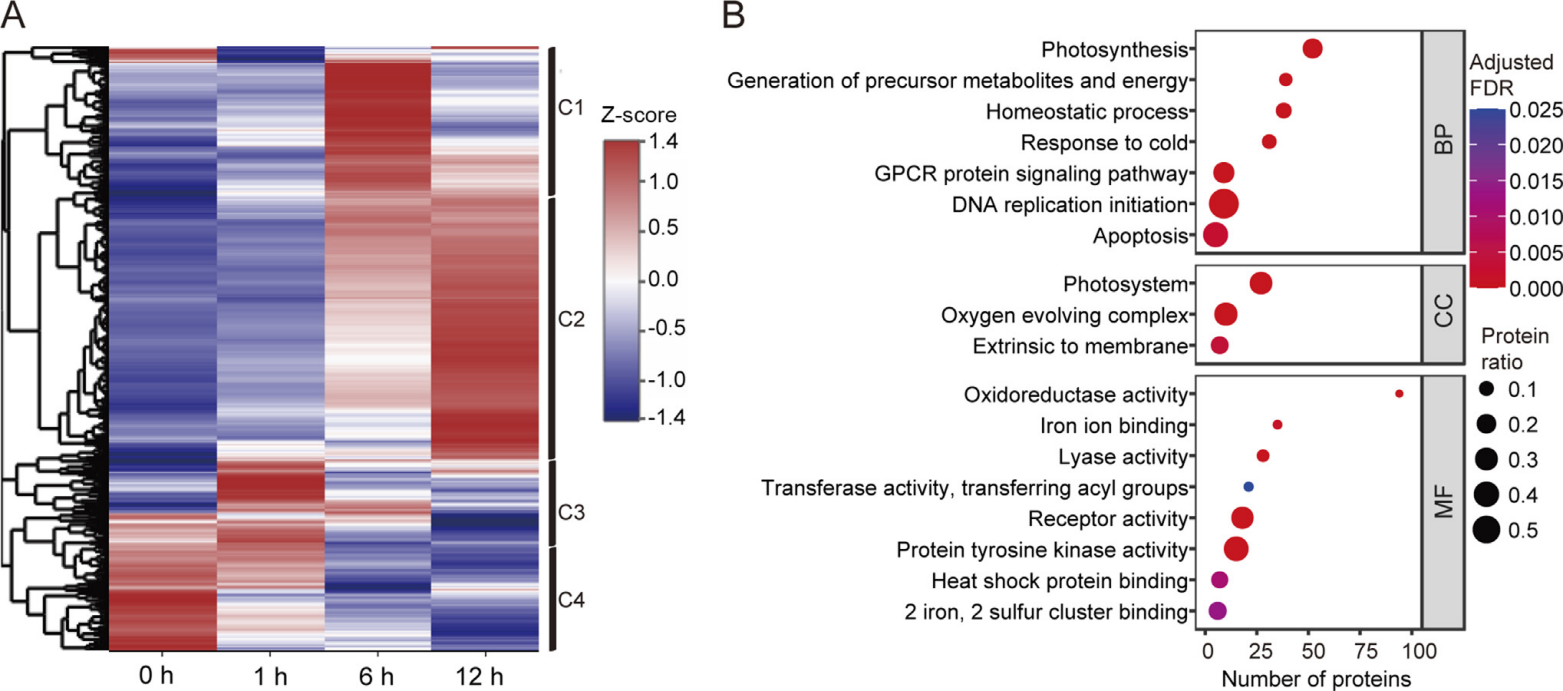
**Proteome dynamics** **A.** Heat map of hierarchical clustering analysis of DEPs. The relative protein abundance (the ratio to 0 h) was normalized by Z-score standardization. Vertical black lines on the right indicate the four clusters (C1–C4) defined based on expression pattern. **B.** GO enrichment analysis of the DEPs identified in this study. Based on GO slim terms all DEPs were assigned to BP, CC, and MF GO categories. Terms that were significantly enriched in DEPs (adjusted FDR ≤ 0.05) are shown. The protein ratio is the ratio of the number of DEPs annotated to a certain GO term (adjusted FDR ≤ 0.05) to the total number of proteins in the B73 maize genome assigned to that term. The horizontal axis indicates the total number of DEPs annotated to each GO term. DEP, differentially expressed protein; BP, biological process; CC, cellular component; MF, molecular function; GPCR protein signaling pathway, G-protein coupled receptor protein signaling pathway.

**Figure 4:**
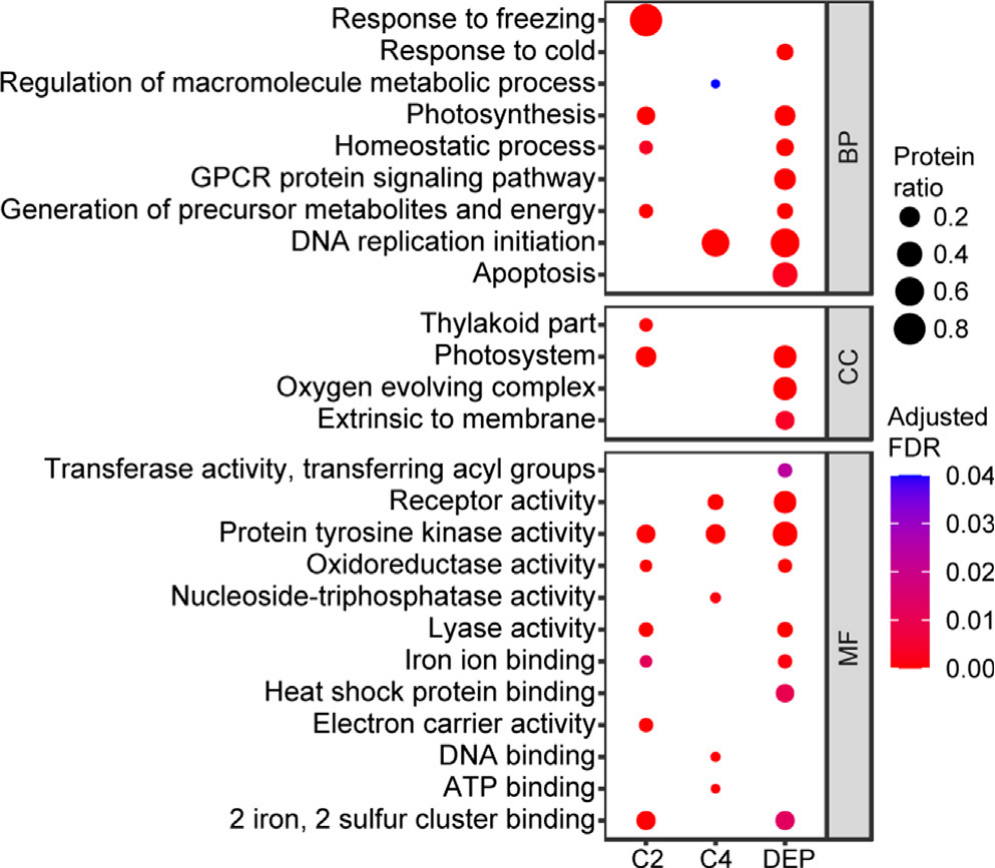
**The enrichment of GO categories in DEPs belonging to cluster 2 and cluster 4** Based on GO slim terms, the DEPs belonging to cluster 2 (continuously upregulated during illumination) and cluster 4 (dramatically downregulated upon illumination) were assigned to BP, CC, and MF GO categories. Terms that were significantly enriched in all DEPs and DEPs belonging to cluster 2 and cluster 4 (adjusted FDR ≤ 0.05) are shown. The protein ratio is the ratio of the number of DEPs annotated to a certain GO term (adjusted FDR ≤ 0.05) to the total number of proteins in the B73 maize genome assigned to that term.

To better understand the molecular mechanism of maize seedling photomorphogenesis, we systematically investigated the proteome dynamics during de-etiolation. We used a strict cutoff criterion, fold change in abundance ≥ 1.50 or ≤ 0.67, to identify proteins with significant changes in abundance, *i.e.*, differentially expressed proteins (DEPs) [34]. To our surprise, only 998 of the 14,168 (7.0%) quantified proteins, representing 980 genes, significantly changed in abundance during the de-etiolation process (Table S1). To reveal the accumulation patterns of DEPs during de-etiolation, we firstly performed hierarchical clustering analysis using the average fold change in intensity ratios. As shown in Figure 3A and Table S5, DEPs were divided into four clusters, with DEPs in clusters 1–4 displaying the highest abundance after 6 h, 12 h, 1 h, and 0 h of illumination, respectively. Nearly half of the proteins were assigned to cluster 2, in which the protein abundances continuously increased with prolonged illumination. Conversely, the abundances of proteins belonging to cluster 4 were dramatically downregulated after illumination. We next performed GO enrichment analysis of all DEPs (Figure 3B). We found that seven biological process (BP) terms, three cellular component (CC) terms, and eight molecular function (MF) terms were highly enriched in DEPs. Of 52 proteins belonging to the BP term of photosynthesis, only 6 (11.5%) proteins significantly increased in abundance after 1 h, while 45 (86.5%) and 50 (96.2%) increased in abundance after 6 h and 12 h, respectively, suggesting that establishment of the photosynthetic machinery mainly occurred after 6 h of illumination. We also performed GO enrichment analysis for DEPs belonging to cluster 2 and cluster 4 (Figure 4). Four BP terms were highly enriched in cluster 2 proteins: response to freezing, photosynthesis, homeostatic process, and generation of precursor metabolites and energy. Numerous studies have shown that there is a complex crosstalk between pathways in response to light and low temperature although the mechanism remains poorly understood. For example, PIF3 and HY5 are key regulators in light response, besides they both play vital roles in response to low temperature in *Arabidopsis* [35], [36], [37]. In the present study, when etiolated maize undergone photomorphogenesis, lots of proteins involved in response to light signals were changed in abundance, which might also play roles in resisting cold stress, so “response to freezing” was enriched in cluster 2 proteins. In contrast, DNA replication initiation and regulation of macromolecule metabolic process were the most highly enriched BP terms for cluster 4 proteins. Though we did not find significantly enriched BP terms containing photoreceptors, we also followed with interest the changes in the abundances of photoreceptors during the de-etiolation process. The abundances of Phytochrome A (PHYA), PHYB, PHYC, and Cryptochrome 2 (CRY2) were sharply downregulated after 12 h of light treatment (Table S1). This is consistent with the previous finding that photoreceptors are activated by light-induced phosphorylation, which eventually initiates their ubiquitination and degradation [17], [38], [39].

### Characterization of phosphorylated peptides

**Figure 5:**
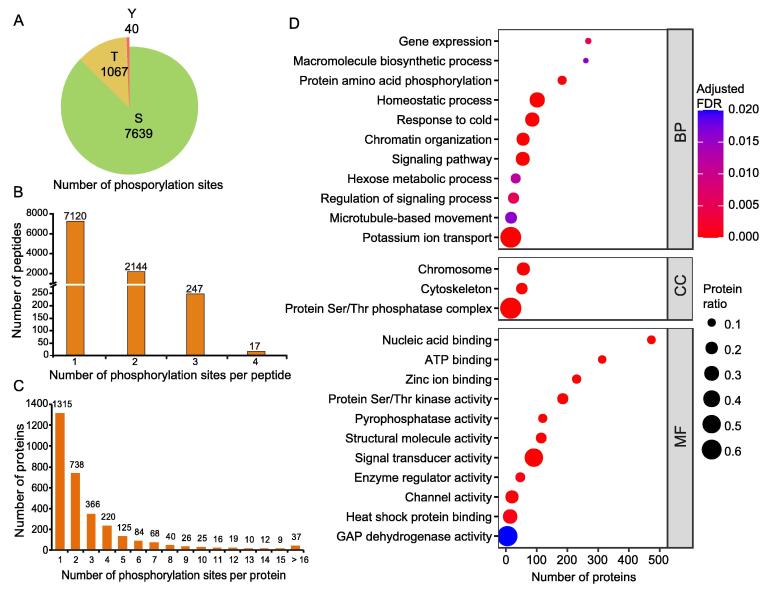
**Overview of the phosphoproteome of de-etiolated maize seedling leaves** **A.** Pie chart showing the number of pS, pT, and pY sites identified in the phosphoproteome. **B.** Distribution of phosphorylation sites per peptide. **C.** Distribution of phosphorylated proteins. **D.** GO enrichment analysis of all phosphorylated proteins identified in this study. Based on GO slim terms all phosphorylated proteins were assigned to BP, CC, and MF GO categories. Terms that were significantly enriched in DEPs (adjusted FDR ≤ 0.05) are shown. The protein ratio is the ratio of the number of the phosphorylated proteins annotated to a certain term (adjusted FDR ≤ 0.05) to the total number of proteins in the B73 maize genome assigned to that term. The horizontal axis indicates the total number of phosphorylated proteins annotated to each GO term. pS, phosphorylated serine; pT, phosphorylated threonine; pY, phosphorylated tyrosine. GAPDH, glyceraldehyde-3-phosphate dehydrogenase. S, serine; T, threonine; Y, tyrosine.

The number of phosphorylation sites per phosphorylated peptide and protein varied greatly. We found 8746 phosphorylation sites in 9528 phosphorylated peptides that matched 3110 phosphorylated proteins (Figure 5). The most abundant phosphorylation site was S (7639, 87.3%), followed by T (1067, 12.2%) and Y (40, 0.5%) (Figure 5A). This suggests that S is the chief site modified by phosphorylation in maize leaves. 7120 (74.7%) peptides contained only one phosphorylation site (Figure 5B; Table S2). Among the phosphorylated proteins, 1057 (34.0%) contained more than 2 phosphorylation sites and 37 contained more than 16 phosphorylation sites (Figure 5C). For instance, the splicing factor PWI (GRMZM2G057646_P03) and cyclophilin (GRMZM2G006107_P02) were hyperphosphorylated, containing 48 and 40 phosphorylation sites, respectively (Table S2).

In the interest of revealing the pathways regulated by phosphorylation during the de-etiolation of etiolated maize seedlings, GO enrichment analysis of all 3110 phosphorylated proteins was performed (Figure 5D). Eleven BP terms were highly enriched in phosphorylated proteins expressed during de-etiolation, such as protein amino acid phosphorylation, signaling pathway, and regulation of signaling process. The highest protein ratio of phosphorylated proteins (the number of phosphorylated proteins annotated to a certain GO term to the total number of proteins in the B73 maize genome assigned to that term) in BP category was observed for the potassium ion transport term (0.67), because of the relatively low total number of proteins (24) assigned to this term. The ratios of phosphorylated proteins in the homeostatic process, response to cold, chromatin organization, and signaling pathway terms (0.31, 0.27, 0.22, and 0.25, respectively) were also relatively high. This indicates that phosphorylation modification may play a crucial role in the regulation of these processes during de-etiolation.

**Figure 6:**
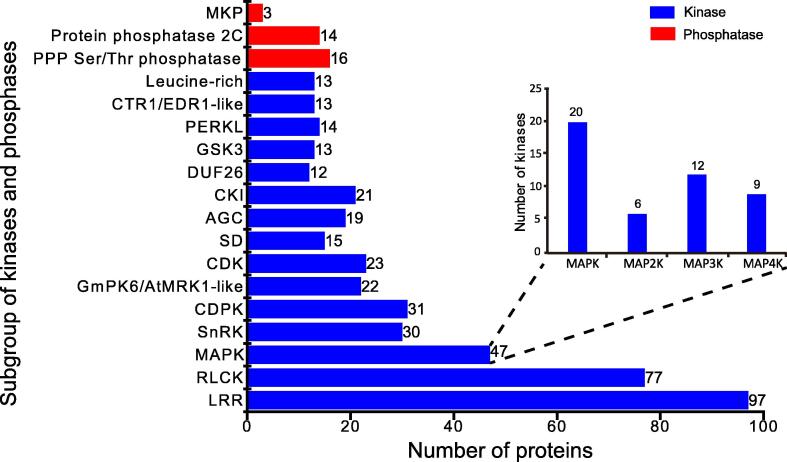
**Classification of the identified kinases and phosphatases** The kinases identified in this study were classified according to the protein kinase classification system described by Lehti-Shiu and Shiu [40], and  phosphatases identified in this study were classified according to ProFITS (http://bioinfo.cau.edu.cn/ProFITS/index.php), ITAK (http://bioinfo.bti.cornell.edu/cgi-bin/itak/index.cgi), and the P3DB database. The kinase families including more than 10 members and the chief phosphatase families are shown. The inset shows the four MAPKs families and the number of members of each family identified in this study. More information about the kinases and phosphatases is shown in Table S6. LRR, leucine rich repeat receptor kinase; RLCK, receptor like cytoplasmic kinase; MAPK, mitogen activated protein kinase; SnRK, SNF1-related protein kinase; CDPK, calcium dependent protein kinase; MRK, MLK/Raf-related protein kinase; CDK, cyclin-dependent kinase; SD, S-domain kinase; AGC, kinase group containing PKA, PKG, and PKC; CKI, casein kinase I; DUF, domain of unknown function; GSK, glycogen synthase kinase; PERKL, protein external response like kinase; CTR, raf-like protein kinase; EDR, enhanced disease resistance; PPP, phosphoprotein phosphatase; MKP, MAP kinase-type phosphatase.

To investigate which proteins bring about changes in phosphorylation during maize leaf de-etiolation, we screened our identified proteins for kinases and phosphatases (Table S6). We quantified 816 kinases and 175 phosphatases, of which 234 kinases and 25 phosphatases were phosphorylated. A total of 543 kinases could be classified into 37 groups according to the protein kinase classification system described by Lehti-Shiu and Shiu [40], and 36 phosphatases were classified into five families according to the ProFITS classification (http://bioinfo.cau.edu.cn/ProFITS/index.php), ITAK (http://bioinfo.bti.cornell.edu/cgi-bin/itak/index.cgi), and the P3DB database. These kinases and phosphatases include two plant-specific kinases, Ser/Thr protein kinase 7 (STN7) and STN8, and one phosphatase, thylakoid associated phosphatase 38 (TAP38)/protein phosphatase 1 (PPH1), which were previously shown to be involved in phosphorylation/dephosphorylation cycles in thylakoids associated with changes in light and diverse other environmental parameters [41]. We also identified 31 calcium-dependent protein kinases (CDPKs), 23 cyclin-dependent kinases (CDKs), 20 mitogen-activated protein kinases (MAPKs), and 27 MAPK cascade kinases (MAP2Ks, MAP3Ks, and MAP4Ks), which play vital roles in transduction pathways (Figure 6; Table S6).

**Figure 7:**
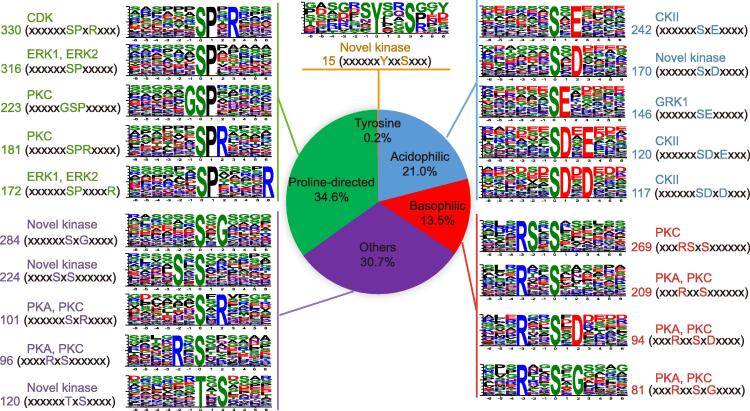
**Motif classification and tabulation of known motifs** Single phosphorylation motifs were identified and overrepresented motifs were extracted. The background was the *Zea mays* AGP v3.28 protein database. The width was set to 13; the significance was set to *P* < 1 × 10^−6^; the occurrence was set to 20 for pS and pT motifs and to 15 for pY motifs. The number shown before the type of motif indicates the number of identified phosphorylated peptides containing the corresponding motif. Sequence logos were generated with Weblogo (http://weblogo.berkeley.edu). Motifs were matched to known kinases using the Phosida motif matcher (http://phosida.de/) and the phosphomotif finder in the HPRD database (http://www.hprd.org/phosphomotif_finder) [42]. ERK, extracellular regulated protein kinase; PKA, protein kinase A; PKC, protein kinase C; CKII, Casein kinase II; GRK I, G protein-coupled receptor kinase I.

Prediction of kinase-motif interactions and analysis of protein quantification/phosphorylation can provide the basis for identifying possible substrates of different kinases. To identify the pathways that are potentially regulated by protein phosphorylation, phosphorylation motifs and the kinases that potentially phosphorylate these sites were also analyzed. Using a method described previously [42], we identified phosphorylation motifs centered on S, T, or Y residues that were overrepresented in phosphorylated peptides using motif-X (http://motif-x.med.harvard.edu) with the *Zea mays* AGPv3.28 protein database as the background. Using stringent criteria for S and T and looser criteria for Y, we identified 64 types of pS-containing motifs (6498 pS sites), 13 types of pT-containing motifs (807 pT sites), and 1 type of pY-containing motifs (15 pY sites) (Table S7). The pS- and pT-containing motifs were classified into three major subgroups, proline-directed (Pro-directed; 34.6%), acidophilic (acidic; 21.0%), and basophilic (basic; 13.5%), as well as others (30.7%) (Figure 7). The kinases that potentially phosphorylate these motifs were identified (see the Materials and methods), and also classified into Pro-directed, acidic, basic, and others based on the types of substrate sequences preferred [43]. The acidic motifs SxE, SDxE, and SDxD, which are phosphorylated by Casein kinase II (CKII), and the basic motifs RSxS, RxxS, RxxSxD, and RxxSxG, which are phosphorylated by protein kinase A (PKA) and protein kinase C (PKC), were identified in our phosphopeptide dataset. The Pro-directed motifs, which are mainly phosphorylated by CDKs and MAPKs, were predominantly found in the phosphorylated peptides containing pS sites (Figure 7; Table S7). The Pro-directed motif SPxR, which is phosphorylated by CDKs, was found in 330 phosphorylated peptides, including the peptides from Retinoblastoma-related protein 1 (RBR1; GRMZM2G003043_P02) which can be phosphorylated by CDKA;1 during the G1 to S phase transition [44]. The Pro-directed motifs SP and SPxxxxR, which are putatively phosphorylated by MAPKs, were found in 316 and 172 phosphorylated peptides, respectively. The dataset of motifs and their corresponding kinases that we have generated can be used to identify new phosphorylation pathways, which will lead to a better understanding of the effect of phosphorylation on maize development.

### Phosphoproteome dynamics in maize leaves

The phosphorylation modification of proteins may be highly complex. Some phosphorylated proteins have multiple phosphorylation sites, and phosphorylation may occur at different phosphorylation sites under different conditions. Thus, there may also be different change patterns of NPLs of peptides during de-etiolation. Here we described the change patterns of HY5 phosphorylation as an example. We identified three isoforms of HY5 (GRMZM2G039828_P01, GRMZM2G137046_P01, and GRMZM2G171912_P01) in maize leaves, and three pS sites were conserved in all three isoforms (Figure S2). Six phosphorylated peptides corresponding to four phosphorylation sites were identified in GRMZM2G039828_P01. We identified two phosphorylated forms of the peptide “TSTTSSLPSSSER”: one was phosphorylated at the fifth S, and the other was phosphorylated at the ninth S; the changes in NPLs were different from each other. Moreover, these two phosphorylated forms of “TSTTSSLPSSSER” were also identified in GRMZM2G171912_P01, which showed very different changes in NPLs compared with those from GRMZM2G039828_P01.

In order to reveal what types of proteins are regulated by phosphorylation in etiolated seedlings exposed to light, we screened for significantly changed phosphorylated peptides (SCPPs) using stringent cutoff criteria (see Materials and methods). In brief, the phosphorylated peptides with a fold change in NPL ≥ 1.50 or ≤ 0.67 were considered significantly changed. The proteins matched by these peptides were considered as proteins with significantly changed phosphorylation (PSCPs). We identified 1475 SCPPs matching 826 PSCPs encoded by 823 genes (Table S2). In fact, the number of PSCPs is likely much higher because many phosphorylated peptides were filtered out because the proteins they corresponded to were not quantified.

**Figure 8:**
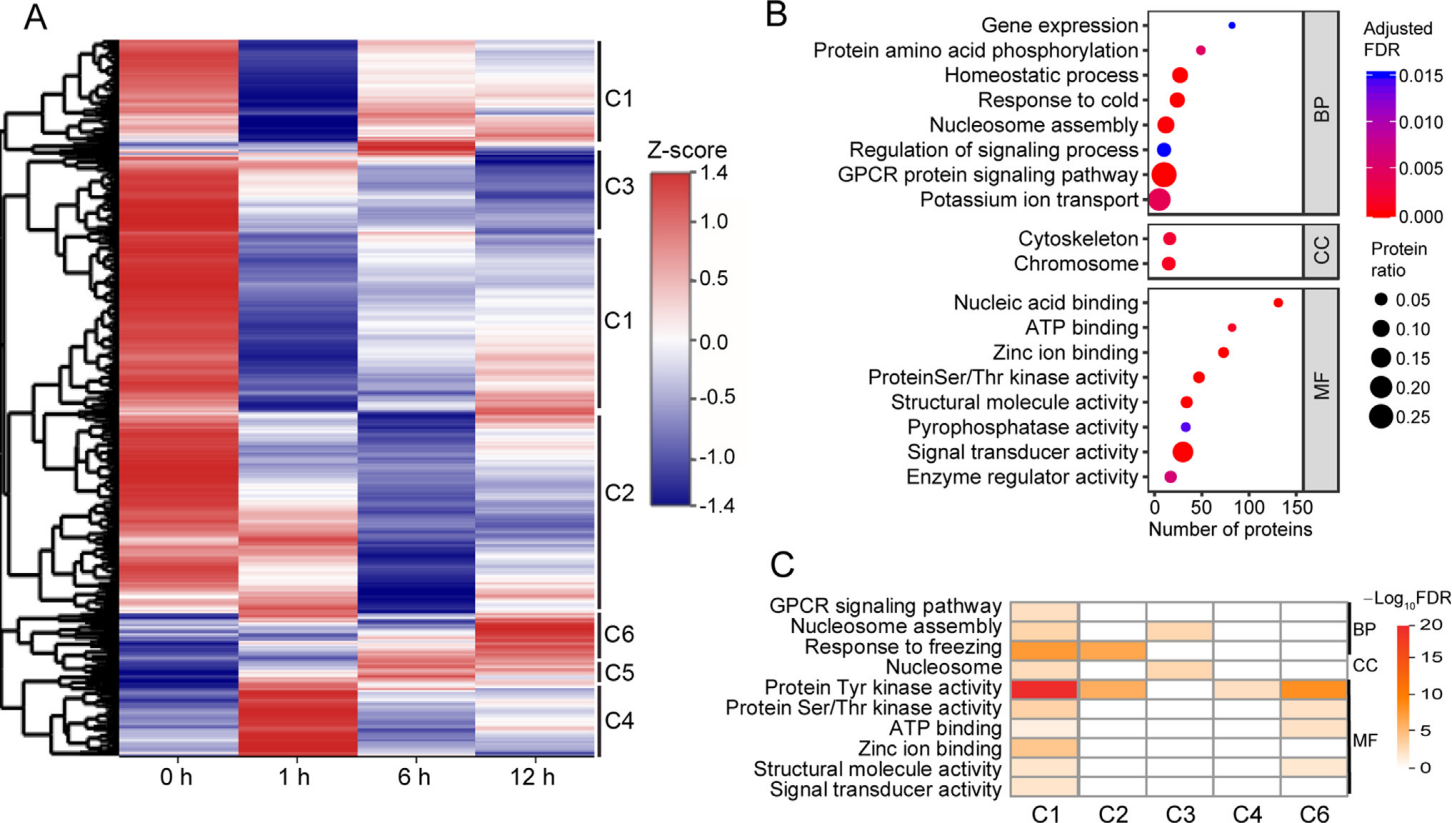
**Phosphoproteome dynamics** **A.** Heat map of hierarchical clustering analysis of SCPPs. The relative phosphorylation level (the ratio to 0 h) was normalized by Z-score standardization. **B.** GO enrichment analysis of PSCPs identified in this study. Based on GO slim terms, all PSCPs were assigned to BP, CC, and MF GO categories. Terms that were significantly enriched in PSCPs (adjusted FDR ≤ 0.05) are shown. The protein ratio is the ratio of the number of PSCPs annotated to a certain term (adjusted FDR ≤ 0.05) to the total number of proteins in the B73 maize genome assigned to that term. The horizontal axis indicates the total number of PSCPs annotated to each GO term. **C.** GO enrichment analysis of PSCPs matched by SCPPs in each cluster in (A). PSCPs corresponding to each cluster were assigned to BP, CC, and MF GO categories. The color of each box indicates the –log_10_ FDR value. Yellow to red represents significant enrichment, and white represents not significant enrichment. SCPP, significantly changed phosphorylated peptide; PSCP, protein with significantly changed phosphorylation; C1–C6, cluster 1–cluster 6.

Next, we performed hierarchical clustering analysis of SCPPs using the average fold change in intensity ratios that were normalized by protein abundance (Figure 8A). SCPPs were assigned to six clusters. Clusters 1–3 contained 79.1% (1166/1475) of SCPPs which showed decreased abundances after illumination. In contrast, SCPPs in clusters 4–6 showed increased abundances after illumination. SCPPs in clusters 1–3 showed lowest abundances after 1 h, 6 h, and 12 h of illumination, respectively, while SCPPs in clusters 4–6 showed highest abundances after 1 h, 6 h, and 12 h of illumination, respectively. We then performed GO enrichment analysis for all PSCPs (Figure 8B; Figure S3). PSCPs were significantly enriched in eight BP terms and two CC terms as shown in Figure 8B. Similar to the proteome enrichment results, PSCPs enriched in cold response pathways were also involved in light signaling, calcium signaling, or protein posttranslational modification, such as non-phototropic hypocotyl 3 (NPH3), 29 kDa ribonucleoprotein A (CP29A), and IQ-domain 17.

We also performed GO enrichment analysis for PSCPs matched by SCPPs in each cluster (Figure 8C; Table S2). For PSCPs in cluster 1, the biggest cluster, three BP, one CC, and six MF terms were enriched, and “protein Tyr kinase activity” was the most significantly enriched term. In cluster 2, PSCPs were enriched in “response to freezing” (BP) and “protein Tyr kinase activity” (MF). PSCPs in cluster 3 were enriched in “nucleosome assembly” (BP) and “nucleosome” (CC). For PSCPs in cluster 4, only one MF term was enriched. In cluster 6, PSCPs were enriched in four MF terms and most of them were kinases. However, no term was significantly enriched for PSCPs in cluster 5. These results revealed that a large number of kinases rapidly changed their phosphorylation statuses after illumination (within 6 h), which might be key regulatory factors for etiolated plants to fast response to light signal and perform photomorphogenesis.

### Transcription factor dynamics

TFs play pivotal roles in the regulation of plant growth and development but have traditionally been difficult to be detected in proteomic analyses because of their low abundances [45], [46]. However, in de-etiolated maize leaves, we identified 724 (28.8%) proteins from 54 TF families comprising 2516 annotated TFs listed in PlantTFDB 3.0 (Table S8). The abundances of 37 out of 498 quantified TFs (7.4%) significantly changed during de-etiolation. Of these TFs, 29 were involved in the MapMan Bin “regulation of transcription”. Consistent with the role of the CONSTANS-like (CoL) protein CoL3 as a positive regulator of photomorphogenesis in *Arabidopsis* [47], we observed that the abundances of CoL4 and CoL5 drastically increased. We also observed dramatic changes in the abundances of one BRI1-EMS suppressor1 (BES1) and two GATA proteins, which are considered to play central roles in the brassinosteroid- and light-signaling pathways [48], [49].

Among all identified TFs, 207 were phosphorylated. Of these phosphorylated TFs, 77 were only identified through the enrichment of phosphorylated peptides, probably due to their low abundances (Figure 2D). The phosphorylation levels of 72 phosphorylated peptides, which matched 48 proteins belonging to 21 TF families, significantly changed during de-etiolation (Figure S4). Interestingly, the phosphorylation levels of two CoL proteins (CoL11 and CoL16) changed during de-etiolation. Therefore, our data suggest that these TFs with significant changes in phosphorylation level might function at higher levels in the hierarchy of gene transcriptional regulation during de-etiolation.

### Phosphorylation plays an essential role in the regulation of light signaling

Seventy-four proteins involved in various light signaling pathways were quantified. Only 13 of these proteins drastically decreased in abundance during de-etiolation. The NPLs of 25 phosphorylation sites in 11 proteins drastically changed (Table S9), indicating that phosphorylation of these sites may play an important role in regulating light signaling pathways.

In *Arabidopsis*, PHYA is the major photoreceptor under far-red light, while PHYB plays a primary role under red or white light with the aid of PHYA, PHYC, and PHYD [50]. In rice, PHYA and PHYB make equal contributions to seedling photomorphogenesis under red light, while both PHYA and PHYC are included in far-red light response [51]. The maize genome has six genes encoding PHY proteins, including two *PHYA* genes (GRMZM2G157727 and GRMZM2G181028), two *PHYB* genes (GRMZM2G092174 and GRMZM2G124532), and two *PHYC* genes (GRMZM2G057935 and GRMZM2G129889). Here we observed that two PHYAs, one PHYB (GRMZM2G092174_P01), and one PHYC (GRMZM2G057935_P01) significantly decreased in abundance during photomorphogenesis. This suggests that these four photoreceptors are likely involved in repressing photomorphogenesis in etiolated seedlings and that the de-etiolation mechanism regulated by *PHY* genes is highly conserved among monocotyledonous plants. It is noteworthy that although the abundance of the PHYB protein (GRMZM2G124532_P03) only decreased slightly in response to light, the NPLs of Thr8, Ser12, Ser49, and Ser76 in this protein drastically increased. In *Arabidopsis*, phosphorylation on Ser86 of PHYB plays an important role in modulating PHY-controlled signaling by accelerating the inactivation of PHYB [52]. Ser76 in maize PHYB (GRMZM2G124532_P03) and Ser86 in AtPHYB are located in the same domain (Figure S5); however, further experiments are needed to confirm whether they have similar functions. Nevertheless, these PTMs in PHYB may play important roles in modulating red or far-red light signaling pathways in maize seedlings just as in *Arabidopsis*.

CRY1 and CRY2 are responsible for photomorphogenesis under blue and UVA light [53], and autophosphorylation is important for their functions [54]. Here we quantified four out of the five maize CRY1 proteins (GRMZM2G024739_P01, GRMZM2G049549_P01, GRMZM2G104262_P01, and GRMZM2G462690_P02) and the single CRY2 protein (GRMZM2G172152_P01). Only CRY2 drastically decreased in abundance, and at the same time the NPLs of Ser480 and Ser483 in CRY2 drastically decreased (Table S9). In *Arabidopsis*, phosphorylation on three serine residues (Ser588, Ser599, and Ser605) in the CRY C-terminal Extension (CCE) domain of CRY2 determines its photosensitivity [55]. Amino acid sequence alignment between the *Arabidopsis* and maize CRY2 proteins revealed that Ser480 of the maize CRY2 protein is located in the CCE domain and corresponds to Ser599 of *Arabidopsis* CRY2 (Figure S6). This suggests that the regulatory mechanism controlling CRY2 photosensitivity is likely conserved among various plant species.

NPH3 is a member of a large family of highly conserved plant-specific proteins that interact with phototropins [56]. Previous studies showed that NPH3 is phosphorylated in dark-grown seedlings; its dephosphorylation is stimulated by blue light and appears to be correlated with phototropism [57], [58]. In *Arabidopsis*, three phosphorylation sites (Ser212, Ser222, and Ser236) on NPH3 were identified by immunoblotting analysis, which were phosphorylated under dark conditions [59]. Here we identified 11 members of the NPH3 family in maize, and the abundance of one of them was significantly downregulated in response to light (Table S9). Moreover, eight of these NPH3 proteins were found to be phosphorylated, and the NPLs of five phosphorylated peptides belonging to three of the eight NPH3 proteins decreased during de-etiolation. For example, 16 phosphorylation sites were identified in one NPH3 protein (GRMZM2G413113_P01), and the NPLs of its two peptides QSPSQNQpSPKpTPSR and WLPDVAPPTpSSSASGR significantly decreased at 6 h. The tendency of reduction in the NPLs of most phosphorylation sites of NPH3 proteins during seedling de-etiolation (Table S9) is in agreement with the deductions of previous studies [58], [59].

### Phosphorylation plays an important role in regulating proteins involved in photosynthetic light reactions

**Figure 9:**
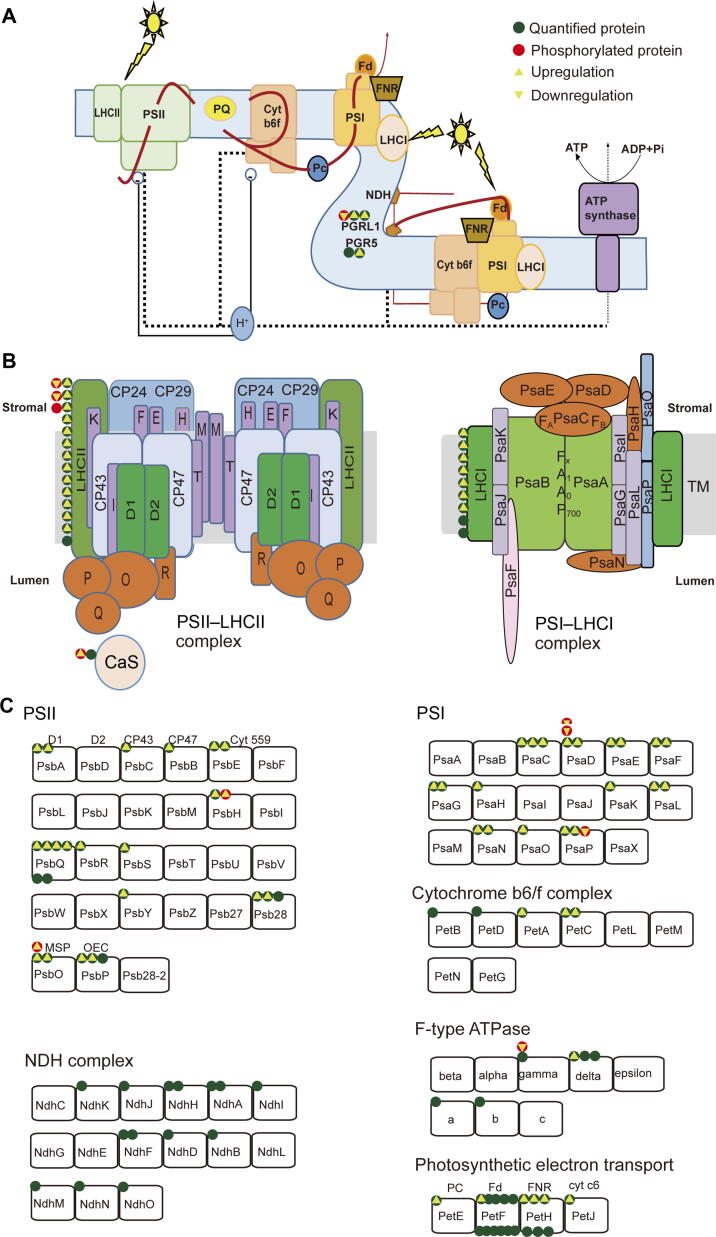
**Phosphorylation of proteins involved in photosynthetic light reaction pathways changes significantly during de-etiolation** This figure was modified from figures in the KEGG photosynthesis pathway (https://www.kegg.jp/kegg-bin/). **A.** The complexes involved in the photosynthetic electron transfer chain. **B.** A model for the assembly of PSII and PSI complexes. **C.** The components in each complex which have been annotated in the KEGG photosynthesis pathway. The green and red circles separately indicate quantified and phosphorylated proteins that function in the light reactions of photosynthesis. The number of circles for each component represents the number of homologs identified in this study. A green circle with an upward triangle indicates that the abundance of the protein was significantly upregulated during de-etiolation; a red circle with an upward or downward triangle indicates that the NPL of the protein was significantly upregulated or downregulated during de-etiolation. NPL, normalized phosphorylation level.

During de-etiolation, proteins involved in photosynthetic pathways had the most dramatic increases in abundance. In previous studies, 13 of 52 significantly changed proteins in rice and 31 of 73 in maize were related to the photosynthetic pathway [24], [25]. Here, we identified 155 proteins involved in photosynthetic light reactions, and 154 of them were quantified (Figure 9; Table S10). Of these proteins, 84 (54.5%), including 28 PSI and 39 PSII subunits, significantly increased in abundance during de-etiolation. For example, CP29 abundance was 2.0-, 6.8-, and 9.4-fold higher in the 1 h, 6 h, and 12 h samples, respectively, than in the 0 h sample.

Eleven proteins involved in photosynthetic light reactions were found to be phosphorylated, and the NPLs of nine proteins, namely four PSII subunits (PsbO-2, PsbH, CP29, and LHCB1.5), two PSI subunits (PsaD-1 and PsaP), one ATP synthase subunit (γ subunit), a protein involved in cyclic electron flow (PGRL1), and a calcium sensing receptor (CaS), drastically changed (Table S10). In particular, the NPL of Thr377 in CaS increased 2.8-fold at 1 h, 13.7-fold at 6 h, and 20.0-fold at 12 h compared with the 0 h sample (Table S10). Sequence alignment indicated that Thr377 of maize CaS corresponds to Thr380 of *Arabidopsis* CaS, which is one of the target sites of the thylakoid protein kinase STN8 (Figure S7). In *Arabidopsis*, CaS is essential for regulating the transcription of photosynthetic electron transport (PET)-related genes, the formation of the PET system, and water use efficiency [60]. Therefore, the drastically increased NPL of Thr377 in CaS might be tightly related to the formation or regulation of the PET system.

### Phosphorylation plays a pivotal role in regulating the activities of enzymes involved in carbon assimilation

**Figure 10:**
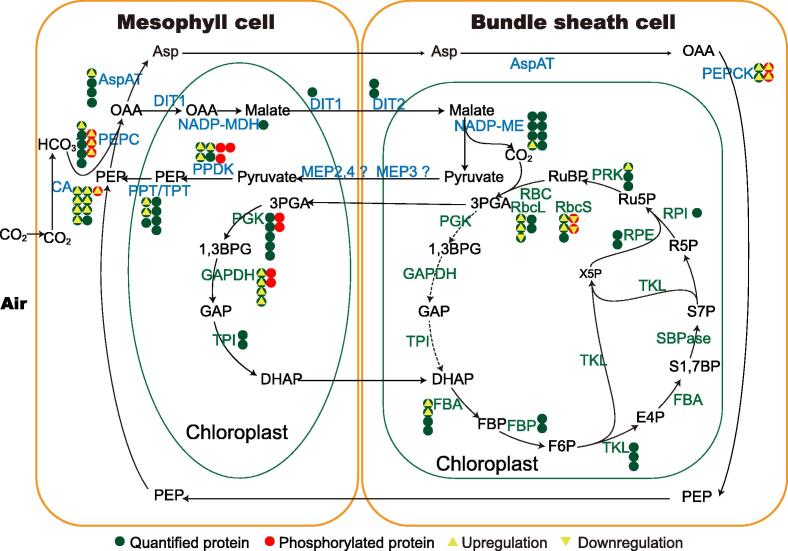
**Phosphorylation of proteins involved inphotosynthetic carbon assimilation changes significantly during de-etiolation** This figure was modified from figures by Majeran et al. [28] and Wingler et al. [64]. Proteins written in blue belong to the C4 pathway, and proteins written in green belong to the Calvin cycle. The green and red circles separately indicate the quantified and phosphorylated proteins that function in photosynthetic carbon assimilation of the C4 plants. The number of circles for each component indicates the number of homologs identified in this study. A green circle with an upward or downward triangle indicates that the abundance of the protein was significantly upregulated or downregulated during de-etiolation; a red circle with an upward or downward triangle indicates that the NPL of the protein was significantly upregulated or downregulated during de-etiolation. CA, carbonic anhydrase; PEPC, phosphoenolpyruvate carboxylase; AspAT, aspartate aminotransferase; DIT, dicarboxylate transporter; NADP-MDH, malate dehydrogenase [NADP]; NADP-ME, NADP-dependent malic enzyme; MEP, envelope protein; PPT, phosphoenolpyruvate/phosphate translocator; PGK, phosphoglycerate kinase; TPI, triosephosphate isomerase; FBA, fructose-bisphosphate aldolase; FBP, fructose-1,6-bisphosphatase; TKL, transketolase; SBPase, seduheptulose bisphosphatase; RPI, ribose-5-phosphate isomerase; RPE, ribulose-phosphate 3-epimerase; PRK, phosphoribulokinase; OAA, oxaloacetic acid; PEP, phosphoenolpyruvate; Asp, aspartate; RuBP, ribulose 1,5-bisphosphate; Ru5P, ribulose 5-P; R5P, ribose 5-P; S7P, sedoheptulose 7-P; S1,7BP, sedoheptulose 1,7-bisphosphate; E4P, erythrose 4-P; F6P, fructose 6-P; FBP, fructose bisphosphate; DHAP, dihydroxyacetone phosphate; GAP, glyceraldehyde 3-phosphate; 1,3BPG, 1,3 biphosphoglycerate; 3PGA, 3-phosphoglycerate.

As a classical C4 plant, both the Calvin cycle and the C4 pathway are active in maize leaves. Here, we identified 87 proteins involved in carbon assimilation, including 44 involved in the Calvin cycle and 43 involved in the C4 pathway (Figure 10; Table S11). Of these proteins, 33 significantly increased in abundance during de-etiolation. Moreover, 16 proteins related to the Calvin cycle or the C4 pathway were phosphorylated, and the NPLs of 21 phosphorylated peptides corresponding to 8 of these proteins significantly changed. These phosphorylated proteins with significant changes in NPL included key enzymes in the Calvin cycle, such as the Rubisco small subunits (RbcS), and key enzymes in the C4 pathway, such as carbonate dehydratase (CA), phosphoenolpyruvate carboxylase (PEPC), and phosphoenolpyruvate carboxykinase (PEPCK).

PEPC is one of the most important C4 photosynthesis enzymes in maize and catalyzes the carboxylation of phosphoenolpyruvate (PEP) to yield oxaloacetate and inorganic phosphate [61]. The NPLs of three Ser sites separately in three PEPC proteins (GRMZM2G069542_P01, GRMZM2G083841_P01, and GRMZM2G473001_P01) were drastically upregulated in response to light at 1 h compared with those at 0 h, and the NPL of Ser15 in GRMZM2G083841_P01 increased 11.4-fold (Table S11). After 6 h of illumination, the NPLs of Ser7 in GRMZM2G069542_P01 and Ser15 in GRMZM2G083841_P01 decreased to a level similar to that of the 0 h sample, which suggests that phosphorylation rapidly adjusts the enzyme activities of PEPC proteins in response to light.

PPDK is one of the key enzymes involved in C4 photosynthesis and plays an important role in regenerating PEP. Moreover, the activity of PPDK may limit the rate of CO_2_ assimilation in the C4 cycle [62]. We previously reported that light intensity regulates PPDK activity by modulating the reversible phosphorylation of Thr527 by the PPDK regulatory protein (PDRP) [63]. The phosphorylation of Thr527 inhibits the enzymatic activity of PPDK. Here, Thr463 in GRMZM2G097457_P02 and Thr462 in GRMZM2G306345_P03, two sites correponding to Thr527, both displayed a tendency of decreased NPLs after illumination (Table S11). This implies that the activity of PPDK was enhanced and the rate of the carbon reaction increased late during de-etiolation. This may be due to the rapid progression of the light reaction after the establishment of the photosynthetic machinery.

In maize leaves, PEPCK is mainly located in bundle sheath cells and participates in CO_2_ concentration by catalyzing the conversion of oxaloacetate to PEP, releasing one CO_2_ [64].The NPLs of a total of eight sites (Ser47, Thr50, Thr51, Ser55, Thr58, Thr59, Ser67, and Thr120) in two PEPCK proteins (GRMZM2G001696_P01 and GRMZM5G870932_P01) drastically changed after illumination (Table S11). The NPLs of Ser67 and Thr120 in GRMZM2G001696_P01 were sharply downregulated, while those of the other six sites in GRMZM2G001696_P01 and/or GRMZM5G870932_P01 were significantly upregulated. One peptide containing four phosphorylation sites (Ser47, Thr51, Thr58, and Thr59) in GRMZM2G001696_P01 showed a 10.0-fold increase in NPL. These complex phosphorylation changes suggest that PEPCK may have intricate regulatory mechanisms in response to light changes.

### Phosphorylation of plasma and plastid proteins plays an important role in metabolism and ion flow during the de-etiolation of etiolated seedlings

Transporters are located on both the plasma and plastid membranes, and the combined activity of all transporters regulates de-etiolation. During the de-etiolation of maize etiolated seedlings, transporters for sugar, auxin, abscisic acid (ABA), cations, and anions are phosphorylated, which affects their activities [23], [24], [30]. To analyze the changes in transporter abundance and phosphorylation during de-etiolation, we screened the de-etiolated maize seedling proteomic and phosphoproteomic datasets for sugar, hormone, ion (especially calcium transporters in the chloroplast), amino acid, and ammonium transporters. Among all 569 quantified transporters, 34 were significantly upregulated and 13 were downregulated in abundance during de-etiolation (Figure S8; Table S12). For example, the abundance of cationic amino acid transporter 9 (CAT9, GRMZM2G139920_P01) at 6 h was 4.8-fold higher than that at 0 h. Two putative SWEET family proteins (GRMZM2G144581_P01 and GRMZM2G111926_P02) and one plastid glucose transporter (pGlcT1, GRMZM2G098011_P01), which are intercellular and chloroplast sugar transporters, respectively [65], [66], were upregulated. In addition, 114 transporter proteins were found to be phosphorylated, and the NPLs of 48 sites in 24 proteins changed significantly (Table S12).

Tonoplast monosaccharide transporter1/2 (TMT1/2) proteins are sugar transporters that are localized on the vacuolar membrane and probably load glucose and sucrose into the vacuole [67]. ERD6 (Early-responsive to dehydration protein 6)-like proteins are involved in the transport of sugars out of the vacuole under certain conditions such as wounding, pathogen attack, senescence, and carbon/nitrogen (C/N)-starvation, and play roles opposite to those of TMT1/2 proteins [68], [69]. In *Arabidopsis*, four phosphorylation sites of TMT2 are located in the central hydrophilic loop, which was previously reported to be more heavily phosphorylated after cold induction, resulting in enhanced TMT activity [70]. ERD6-like can also be phosphorylated at Ser residues in *Arabidopsis* [69]. In the present study, the NPLs of four Ser sites (Ser276, Ser280, Ser286, and Ser320) in TMT2 (GRMZM2G083173_P01) were downregulated, while the NPL of Ser55 in the ERD6-like protein (GRMZM2G097768_P01) was upregulated (Table S12). However, no change in protein abundance was observed in both Schulze’s and our study (Table S12) [70]. This may be because the regulation of transporter activity by phosphorylation may allow more rapid regulation of sugar transport than changing protein abundance.

Metal ions are critical cofactors for legion chloroplast proteins involved in photosynthesis (Ca^2+^, Mg^2+^, Mn^2+^, and Fe^2+^) and oxidative stress detoxification (Cu^2+^, Zn^2+^, and Fe^2+^), and H^+^-coupled ATPases are important for chloroplast biogenesis. The activities of metal ion transporters control the concentration of ions in specific locations. To understand whether the activities of these transporters are potentially regulated by phosphorylation, we screened for phosphorylation of these cation and proton pumps. We found that the NPLs of Ser82 in potassium transporter KUP12 (GRMZM2G036916_P01) and Thr881 in autoinhibited H^+^-ATPase isoform 2 (AHA2, GRMZM2G019404_P01) were significantly upregulated after 6 h of illumination (Table S12). In addition, the NPLs of Ser298 in CAX interacting protein 4 (CXIP4, GRMZM2G048257_P01) and Ser718 and Ser726 in KUP7 (GRMZM2G139931_P01) sharply reduced after 1 h of illumination (Table S12). The phosphorylation modifications identified in the present study could provide guidance for studying the functional regulation of these transporters by PTMs.

## Conclusion

The transition from etiolation to de-etiolation is a very complicated process, during which plants need to quickly respond to light signals and rapidly mobilize photomorphogenesis to complete the formation of the photosynthetic system and initiate photosynthetic reactions. In the present study, we have provided the most comprehensive dynamic analyses of protein abundance and phosphorylation in de-etiolated maize leaves to date.

Among the 14,168 quantified proteins in proteome, only 998 proteins (7.0%), including 37 TFs, significantly changed in abundance during the de-etiolation process; these proteins included nearly all those previously shown to change in abundance at similar developmental stages [23], [25], [71]. In contrast, 26.6% of phosphorylated proteins, especially those involved in gene expression, protein amino acid phosphorylation, and homeostatic process pathways, significantly changed in phosphorylation level. In addition, 25.3% (2408/9528) of identified phosphorylated peptides contained more than one phosphorylation sites; 1057 (34.0%) phosphorylated proteins containing three or more phosphorylation sites, and 128 of them contained ten or more phosphorylation sites. These phosphorylation sites may regulate different aspects of protein functions by activating or inhibiting protein activities, which may in turn regulate the functions of these proteins in different pathways. Moreover, these effects may be enhanced by phosphorylation at multiple sites in the same protein.

Our data suggest that the regulation of PTM levels on proteins might be more efficient than the regulation of protein abundance for plants to adapt to changing environments. Reversible PTMs allow plants to rapidly respond to internal and external cues. In addition, PTM is more economical in terms of energy use than *de novo* protein synthesis, which involves several steps from the initiation of gene transcription to the formation of a mature protein; only a little energy (ATP or GTP) is needed to add or to remove a functional group (PTM) on a protein in order to change its physical and chemical properties. Therefore, the study of protein PTMs is important to fully explore the mechanisms of plant adaptation to environmental changes.

## Materials and methods

### Plant material and sample collection

The maize inbred line B73 was used in this study. The seedlings were planted and samples were collected as described previously [32]. Under the same conditions, two biological replicates were performed and the first seedling leaves from each replicate were rapidly sampled. All samples were frozen in liquid nitrogen and stored at −80 °C until further use.

### Protein extraction

Total proteins were extracted from maize seedling leaves using a 10% (w/v) trichloroacetic acid (TCA)/acetone solution as described previously [32]. The protein concentration of each sample was determined using the 2-D Quant kit (GE Healthcare, Boston, MA). Protein samples were stored at −80 °C for further experiments.

### Sample preparation

Protein extracts of two sets of maize samples (0 h, 1 h, 6 h, and 12 h, ~ 5 mg each) were digested by trypsin as previously described [32]. According to the manufacturer’s instructions, samples were labeled with iTRAQ 4plex reagent (ABSciex, MA) and then combined.

### SCX chromatography

Total iTRAQ-labeled lysate was solubilized in buffer A (5 mM KH_2_PO_4_, 25% acetonitrile, pH 3.0) and separated on a PolySULFOETHYL A column (4.6 mm ID × 100 mm, 5 µm, 300 Å; Poly LC Inc, Columbia, MD) with flow rate of 1 ml/min using a linear gradient of 0% buffer B (5 mM KH_2_PO_4_, 25% acetonitrile, 400 mM KCl, pH 3.0) to 100% buffer B over 40 min. A Gilson system composed of 306 pumps, an 805 manometric module, an 811C dynamic mixer, and a UV/VIS-155 detector was used. The sample fractions were collected every minute and dried.

### Basic reverse phase HPLC

For general proteomics, the iTRAQ-labeled total lysate or the selected SCX fractions described above were solubilized and separated as previously described [72]. For phosphoproteomics, ammonium formate was switched to ammonium for every 6th fractions from fractions 10–45 to generate a total of 6 pooled fractions. The pooled fractions were subsequently dried under vacuum and subjected to the enrichment of phosphorylated peptides using the IMAC method or TiO_2_.

### Enrichment of phosphorylated peptides using IMAC and TiO_2_

The protocol for enrichment of phosphorylated peptides using IMAC was adapted from Mertins et al. [73] with modifications. The procedure for enrichment of phosphorylated peptides using TiO_2_ was adapted from Wilson-Grady et al. [74] with modifications. The enriched phosphorylated peptides were further desalted using an Empore C18 (Catalog No. 2215; 3M, Minneapolis, MN) prior to HPLC-MS/MS analysis.

### HPLC-MS/MS

A Dionex RSLC system interfaced with QExactive HF (ThermoFisher Scientific, San Jose, CA) was used to carried out HPLC-MS/MS primarily. Due to instrument availability, 2D-HPLC-MS/MS and phosphor-proteomic samples were analyzed using a Dionex RSLC system interfaced with a Velos LTQ Orbitrap ETD (ThermoFisher Scientific) as described previously [32]. Mass spectrometry data was acquired using a data-dependent acquisition procedure with a cyclic series of a full scan acquired in the Orbitrap with a resolution of 120,000 (QExactive HF) or 60,000 (VELSO LTQ Orbitrap ETD), followed by MS/MS scans (38% of relative collision energy in the HCD cell) of 10 most intense ions with a repeat count of one and the dynamic exclusion duration of 30 s and scanned out in the Orbitrap with a resolution of 30,000 (QExactive HF) or 15000 (VELOS LTQ Orbitrap ETD) with low mass set at 110 amu.

### Data analysis

The HPLC-MS/MS data from each experiment were searched as described previously [32]. For proteins identified only in 2D-HPLC-MS/MS, the average ratio of two biological replicates was used to represent the final fold change at each time point, while for proteins identified only in 3D-HPLC-MS/MS or both in 2D- and 3D-HPLC-MS/MS, the ratios from 3D-HPLC-MS/MS were used. The proteins were considered significantly changed with a fold change ≥ 1.50 or ≤ 0.67.

### Database searching with the phosphoproteome data

The HPLC-MSMS data were searched in MUDPIT style against the *Zea mays* database using MASCOT (version 2.3 MatrixScience, UK) or MSAmanda (version 1.4.14.3866 for PD1.4.1.14) [75] in the Proteome Discoverer (version 1.4.1.14; ThermoFisher Scientific, Bremen, Germany) environment. For both search engines, oxidation of methionine and phosphorylation on serine, threonine, and tyrosine were set as variable modifications. MASCOT and MSAmanda results were combined for reporting. Percolator was used to validate PTMs. Only top hit peptides with FDR < 0.01 (based on PEP score) were included in the final results. Phosphorylation sites were localized using PhosphoRS 3.1 (implemented in Proteome Discoverer 1.4.1.14). The ratios of phosphorylated peptides between the 0 h control and different time points were calculated using reporter ion intensities. The data were then normalized to general normalization factors determined from the median of high-confidence spectra identified  from HPLC-MS/MS results prior to enrichment (fractionation-phospho-peptide enrichment) or general proteomic data (phospho-peptide enrichment from the total lysate) as described in “the Data analysis” section of the Materials and methods. For the phosphorylated peptides only quantified in one experiment, the ratios were used to represent the final fold change at each time point, while for the peptides quantified more than twice, the mean value of the ratios from all replicates was used as the final fold change. The phosphorylated peptides with a fold change ≥ 1.50 or ≤ 0.67 were considered significantly regulated.

### SDS-PAGE and immunoblotting

Protein expression and phosphorylation were assessed using standard Western blotting protocols described by Chen and colleagues [63]. Blots were probed with rabbit polyclonal anti-AtpB, anti-PsaD, anti-PsbC, anti-RA, anti-RbcL, anti-RbcS, and anti-UGPase antibodies (Agrisera Antibodies, Vännäs, Sweden) and an anti-plant-actin rabbit polyclonal antibody (EasyBio, Beijing, China). The rabbit polyclonal anti-PPDK and anti-PEPCK antibodies were prepared by our laboratory.

### Motif and kinase-phosphatase analyses

Sequences of phosphorylated peptides were extended to 13 aa with a central S, T, or Y using the *Zea mays* database (V3.28) [42]. Pre-aligned peptides were submitted to the Motif-X algorithm (http://motif-x.med.harvard.edu/). Sites that were located at the N- or C-terminus and thus could not be extended to 13 aa were excluded. The significance was set to *P* < 1 × 10^−6^, and the occurrence was set to 20 for pS and pT motifs and to 15 for pY motifs. Motifs were classified as proline-directed, acidophilic, basophilic, and other as described previously [42]. Sequence logos were generated with Weblogo (http://weblogo.berkeley.edu). All proteins identified in this study were screened for kinases and phosphatases using the GO accession numbers GO: 0016301 for kinases and GO: 0016791 for phosphatases. Kinases were classified according to the protein kinase classification system described by Lehti-Shiu and Shiu [40] and phosphatases were classified according to the maize databases ProFITS (http://bioinfo.cau.edu.cn/ProFITS/index.php), ITAK (http://bioinfo.bti.cornell.edu/cgi-bin/itak/index.cgi), and P3DB (http://p3db.org/).

### Bioinformatics analyses

Protein sequences were obtained from EnsemblPlants (http://plants.ensembl.org/index.html). The functional annotations of proteins were performed using the MapMan (*Zea Mays* genome release 1.1) and Ensemble Plant (AGPv3) databases.

Hierarchical clustering of proteins was done in R (version 3.4.3; https://www.r-project.org) using the heatmaply method from the heatmaply package (version 0.14.1). GO enrichment analyses of proteins and phosphorylated proteins were performed based on agriGO v2.0 (http://systemsbiology.cau.edu.cn/agriGOv2/index.php) with the B73 maize genome V3.3 as the background. Significantly enriched terms in the BP, CC, and MF GO categories were plotted using ggplot from the ggplot2 package (version 2.2.1; http://ggplot2.tidyverse.org) in R (version 3.4.3; https://www.r-project.org).

### Sequence analysis

Sequences of HY5, PHYB, CRY2, and CaS from *Arabidopsis* and maize were downloaded from the Phytozome database (https://phytozome.jgi.doe.gov/pz/portal), and sequence alignment was done using BioEdit software.

## Data availability

These mass spectrometry proteomics and phosphoproteomics data have been submitted to the ProteomeXchange Consortium (ProteomeXchange: PXD012897), and are publicly accessible at http://proteomecentral.proteomexchange.org/cgi/GetDataset.

## CRediT author statement

**Zhi-Fang Gao:** Conceptualization, Formal analysis, Visualization, Writing - original draft, Writing - review & editing. **Zhuo Shen:** Conceptualization, Data curation, Methodology. **Qing Chao:** Conceptualization, Writing - review & editing. **Zhen Yan:** Validation, Visualization. **Xuan-Liang Ge:** Data curation, Formal analysis. **Tiancong Lu:** Data curation, Resources. Haiyan Zheng: Investigation, Software. **Haiyan Zheng:** Investigation, Software. **Chun-Rong Qian:** Conceptualization, Writing - review & editing. **Bai-Chen Wang:** Conceptualization, Funding acquisition, Writing - review & editing. All authors read and approved the final manuscript.

## Competing interests

The authors have declared no competing interests.

## Supplementary material

Supplementary Figure S1 **Comparisons between the 2D proteome and 3D proteome.** The protein abundance data from the 3D proteome analysis were compared with the data from the first and second replicates of the 2D proteome analysis. The Y-axis is the ratio of 1 h/0 h, 6 h/0 h and 12 h/0 h identified in 3D proteome analysis and the X-axis is the ratio of 1 h/0 h, 6 h/0 h and 12 h/0 h identified in the 2D proteome analysis. The Pearson correlation coefficient (r) and P value are shown in the upper-left corner of each plot.

Supplementary Figure S2 **The phosphorylation of three HY5 isoforms in maize**. Peptides corresponding to three HY5 proteins that contain phosphorylation sites identified in this study are shown at the top of the figure. The “S” and “T” residues highlighted by yellow boxes are the phosphorylation sites, and the S residues are conserved in all three peptides. The phosphorylated peptides identified by HPLC-MS/MS after enrichment using IMAC and TiO2 are shown below the sequence alignment, and the red S and T residues are the phosphorylation sites. The bar graphs to the right of each peptide show the fold-changes (FC) in NPL after illumination for 1, 6, and 12 hours. The red asterisks indicate the main phosphorylation sites. S, serine; T, threonine; NPL, normalized phosphorylation level.

Supplementary Figure S3 **Enrichment analysis of all phosphoproteins and the phosphoproteins with significantly changed phosphorylation**. Based on GO slim terms, all identified phosphoproteins (Total) and the phosphoproteins significantly changed in phosphorylation level (with altered phosphorylation level) were assigned to biological process (BP), cellular component (CC) and molecular function (MF) GO categories. Terms that were significantly enriched in phosphoproteins and differentially phosphorylated phosphoproteins (adjusted FDR ≤ 0.05) are shown. The protein ratio is the ratio of the number of phosphoproteins or differentially phosphorylated phosphoproteins annotated to a certain term (adjusted FDR ≤ 0.05) to the total number of proteins in the B73 maize genome assigned to that term. GPCR protein signaling pathway, G-protein coupled receptor protein signaling pathway; GAPDH, glyceraldehyde-3-phosphate dehydrogenase.

Supplementary Figure S4 **The dynamics of the TFs identified in the proteome and phosphoproteome**. **A**. The number of TFs in 17 families with significant changes in protein abundance are shown. **B**. Heat map of the TFs that significantly changed in abundance during de-etiolation. The relatively protein abundance (the ratio to 0 h) was normalized by z-score standardization. FC: fold changes. **C**. The number of TFs in 21 families with significant changes in normalized phosphorylation levels. **D.** Heat map of the TFs with significant changes in normalized phosphorylation levels during de-etiolation. The relatively phosphorylation level of each significantly changed phosphopeptide was normalized by z-score standardization. FC: fold changes. TCP, teosinte branched1, cycloidea, and pcf family; C3H, TFs comprising motif with CCCH; Bzip, basic leucine zipper; B3, TFs containing B3 domain; C2H2, cysteine 2 histidine 2; CO, constans; HB, homeobox; Bhlh, basic helix-loop-helix; HD-ZIP, homeodomain-leucine zipper; NAC, NAC domain-containing protein; BES1, BRI1-EMS suppressor1; G2, golden2; TALE, three amino acid loop extension; NF-YC, nuclear factor Y subunit C; ZF-HD, zinc finger homeodomain; GeBP, glabra1 enhancer binding protein; ARF, auxin response factor; CAMFA, calmodulin-binding transcription activator; NF-YB, nuclear factor Y subunit B; AP2, apetala2; CPP, cystein-rich polycomb-like protein; GRF, growth-regulating factor; CPRF, common plant regulatory factors; ATHB, Arabidopsis thaliana homeobox; LHY, late elongated hypocotyl; ABF, ABRE binding factor; LEC, leafy cotyledon; IDD, indeterminate(ID)-domain; KNAT, knotted-like homeobox of Arabidopsis thaliana; ATRL, Arabidopsis rad-like; ZFN, zinc finger protein; BEL, bell; GRPI, gbfs pro-rich region-interacting factor; COL, constans-like.

Supplementary Figure S5 **Alignment of the AtPHYB and ZmPHYB proteins.** The amino acid sequences encoded by AtPHYB (AT2G18790.1) and two ZmPHYBs (GRMZM2G092174_P01 and GRMZM2G124532_P03) were aligned. The “S” residues outlined in red boxes are the phosphorylation sites Ser86 identified in *Arabidopsis* and Ser76 identified in *Zea mays*.

Supplementary Figure S6 **Alignment of the AtCRY2 and ZmCRY2 proteins.** The amino acid sequences encoded by AtCRY2 (AT1G04400.2) and two ZmCRY2 transcripts (GRMZM2G172152_T01 and GRMZM2G172152_T02) were aligned. The “S” residues outlined in red boxes are the phosphorylation sites identified in *Arabidopsis* and *Zea mays*. The red asterisks indicate the conserved phosphorylation sites.

Supplementary Figure S7 **Alignment of the AtC****a****S and ZmC****a****S proteins**. The amino acid sequences of AtCaS (AT5G23060.1) and ZmCaS (GRMZM2G122715_T02) were aligned. The “T” outlined by a red box is the conserved phosphorylation site identified in both the *Arabidopsis* and *Zea mays* proteins.

Supplementary Figure S8 **The dynamics of transporters with significant changes in protein abundance during de-etiolation.** Heat map showing the hierarchical clustering of transporters with significant changes in abundance. The relatively protein abundance (the ratio to 0 h) was normalized by z-score standardization. FC, fold changes.
