# Supplementary figures and images for: Large-scale Proteomic and Phosphoproteomic Analyses of Maize Seedling Leaves During De-etiolation
Source: Genomics Proteomics Bioinformatics. 2020 Dec 30;18(4):397–414. doi: 10.1016/j.gpb.2020.12.004 (PMC8242269; doi:10.1016/j.gpb.2020.12.004)

Replicate 1

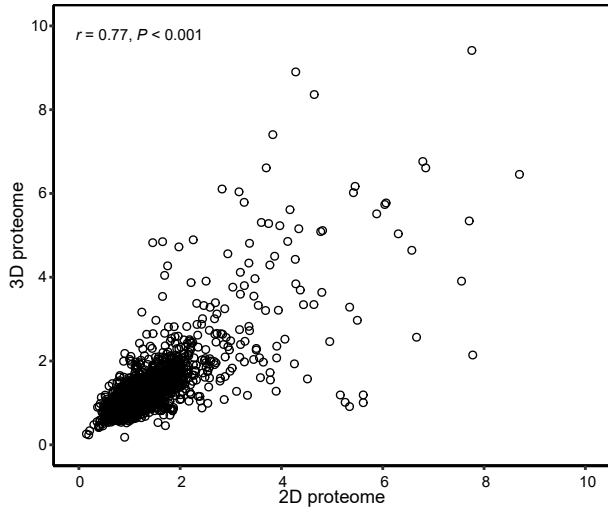

Replicate 2

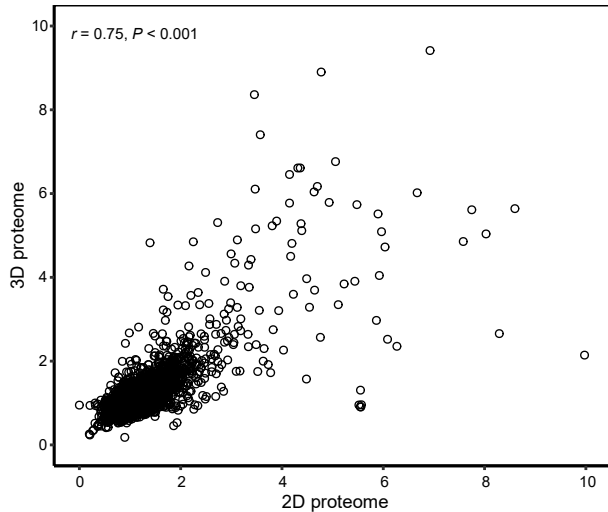

Supplement: Supplementary Figure S1 — Comparisons between the 2D proteome and 3D proteome. The protein abundance data from the 3D proteome analysis were compared with the data from the first and second replicates of the 2D proteome analysis. The Y-axis is the ratio of 1 h/0 h, 6 h/0 h and 12 h/0 h identified in 3D proteome analysis and the X-axis is the ratio of 1 h/0 h, 6 h/0 h and 12 h/0 h identified in the 2D proteome analysis. The Pearson correlation coefficient (r) and P value are shown in the upper-left corner of each plot. [file mmc1.pdf]

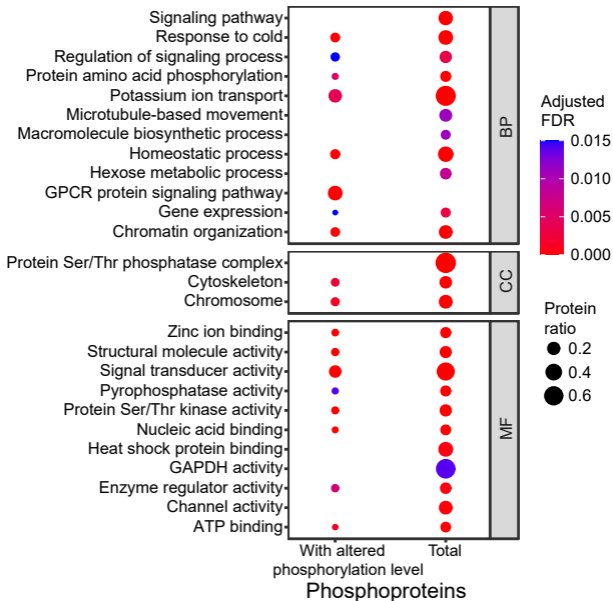

Supplement: Supplementary Figure S3 — Enrichment analysis of all phosphoproteins and the phosphoproteins with significantly changed phosphorylation. Based on GO slim terms, all identified phosphoproteins (Total) and the phosphoproteins significantly changed in phosphorylation level (with altered phosphorylation level) were assigned to biological process (BP), cellular component (CC) and molecular function (MF) GO categories. Terms that were significantly enriched in phosphoproteins and differentially phosphorylated phosphoproteins (adjusted FDR ≤ 0.05) are shown. The protein ratio is the ratio of the number of phosphoproteins or differentially phosphorylated phosphoproteins annotated to a certain term (adjusted FDR ≤ 0.05) to the total number of proteins in the B73 maize genome assigned to that term. GPCR protein signaling pathway, G-protein coupled receptor protein signaling pathway; GAPDH, glyceraldehyde-3-phosphate dehydrogenase. [file mmc3.pdf]

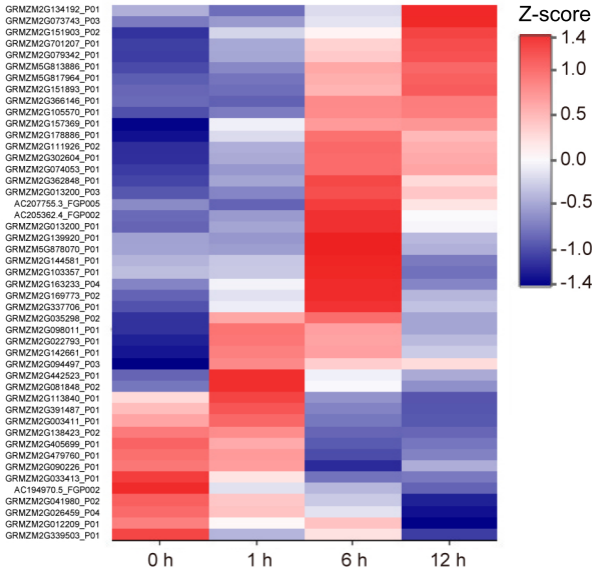

Supplement: Supplementary Figure S8 — The dynamics of transporters with significant changes in protein abundance during de-etiolation. Heat map showing the hierarchical clustering of transporters with significant changes in abundance. The relatively protein abundance (the ratio to 0 h) was normalized by z-score standardization. FC, fold changes. [file mmc8.pdf]
