# Supplementary material for: Large-scale Proteomic and Phosphoproteomic Analyses of Maize Seedling Leaves During De-etiolation
Source: Genomics Proteomics Bioinformatics. 2020 Dec 30;18(4):397–414. doi: 10.1016/j.gpb.2020.12.004 (PMC8242269; doi:10.1016/j.gpb.2020.12.004)
Supplement: Supplementary Figure S2 — The phosphorylation of three HY5 isoforms in maize. Peptides corresponding to three HY5 proteins that contain phosphorylation sites identified in this study are shown at the top of the figure. The “S” and “T” residues highlighted by yellow boxes are the phosphorylation sites, and the S residues are conserved in all three peptides. The phosphorylated peptides identified by HPLC-MS/MS after enrichment using IMAC and TiO2 are shown below the sequence alignment, and the red S and T residues are the phosphorylation sites. The bar graphs to the right of each peptide show the fold-changes (FC) in NPL after illumination for 1, 6, and 12 hours. The red asterisks indicate the main phosphorylation sites. S, serine; T, threonine; NPL, normalized phosphorylation level. [file mmc2.pdf]

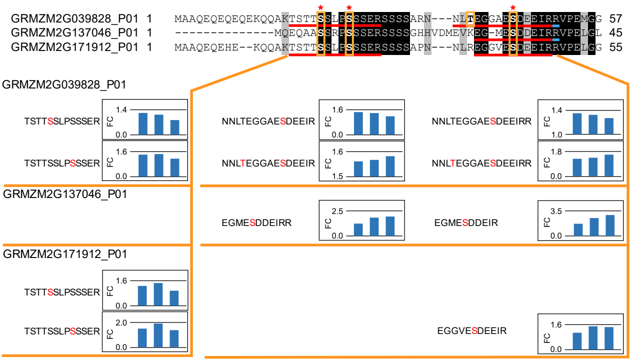
GRMZM2G039828_P01 1 MAAQEQEQEQEKQQAKTSTTSSLPSSSERSSSSARN---NLTEGGAESDEEIRRVPEMGG 57
GRMZM2G137046_P01 1 --------------MQEQAASSRPSSSERSSSSGHHVDMEVKEG-MESDDEIRRVPELGL 45
GRMZM2G171912_P01 1 MAAQEQEHE--KQQAKTSTTSSLPSSSERSSSSAPN---NLREGGVESDEEIRRVPELGG 55
GRMZM2G039828_P01
TSTTSSLPSSSER
TSTTSSLPSSSER
NNLTEGGAESDEEIR
NNLTEGGAESDEEIR
NNLTEGGAESDEEIRR
NNLTEGGAESDEEIRR
GRMZM2G137046_P01
EGMESDDEIRR
EGMESDDEIR
GRMZM2G171912_P01
TSTTSSLPSSSER
TSTTSSLPSSSER
EGGVESDEEIR
FC
