# Supplementary material for: Large-scale Proteomic and Phosphoproteomic Analyses of Maize Seedling Leaves During De-etiolation
Source: Genomics Proteomics Bioinformatics. 2020 Dec 30;18(4):397–414. doi: 10.1016/j.gpb.2020.12.004 (PMC8242269; doi:10.1016/j.gpb.2020.12.004)
Supplement: Supplementary Figure S4 — The dynamics of the TFs identified in the proteome and phosphoproteome. A. The number of TFs in 17 families with significant changes in protein abundance are shown. B. Heat map of the TFs that significantly changed in abundance during de-etiolation. The relatively protein abundance (the ratio to 0 h) was normalized by z-score standardization. FC: fold changes. C. The number of TFs in 21 families with significant changes in normalized phosphorylation levels. D. Heat map of the TFs with significant changes in normalized phosphorylation levels during de-etiolation. The relatively phosphorylation level of each significantly changed phosphopeptide was normalized by z-score standardization. FC: fold changes. TCP, teosinte branched1, cycloidea, and pcf family; C3H, TFs comprising motif with CCCH; Bzip, basic leucine zipper; B3, TFs containing B3 domain; C2H2, cysteine 2 histidine 2; CO, constans; HB, homeobox; Bhlh, basic helix-loop-helix; HD-ZIP, homeodomain-leucine zipper; NAC, NAC domain-containing protein; BES1, BRI1-EMS suppressor1; G2, golden2; TALE, three amino acid loop extension; NF-YC, nuclear factor Y subunit C; ZF-HD, zinc finger homeodomain; GeBP, glabra1 enhancer binding protein; ARF, auxin response factor; CAMFA, calmodulin-binding transcription activator; NF-YB, nuclear factor Y subunit B; AP2, apetala2; CPP, cystein-rich polycomb-like protein; GRF, growth-regulating factor; CPRF, common plant regulatory factors; ATHB, Arabidopsis thaliana homeobox; LHY, late elongated hypocotyl; ABF, ABRE binding factor; LEC, leafy cotyledon; IDD, indeterminate(ID)-domain; KNAT, knotted-like homeobox of Arabidopsis thaliana; ATRL, Arabidopsis rad-like; ZFN, zinc finger protein; BEL, bell; GRPI, gbfs pro-rich region-interacting factor; COL, constans-like. [file mmc4.pdf]

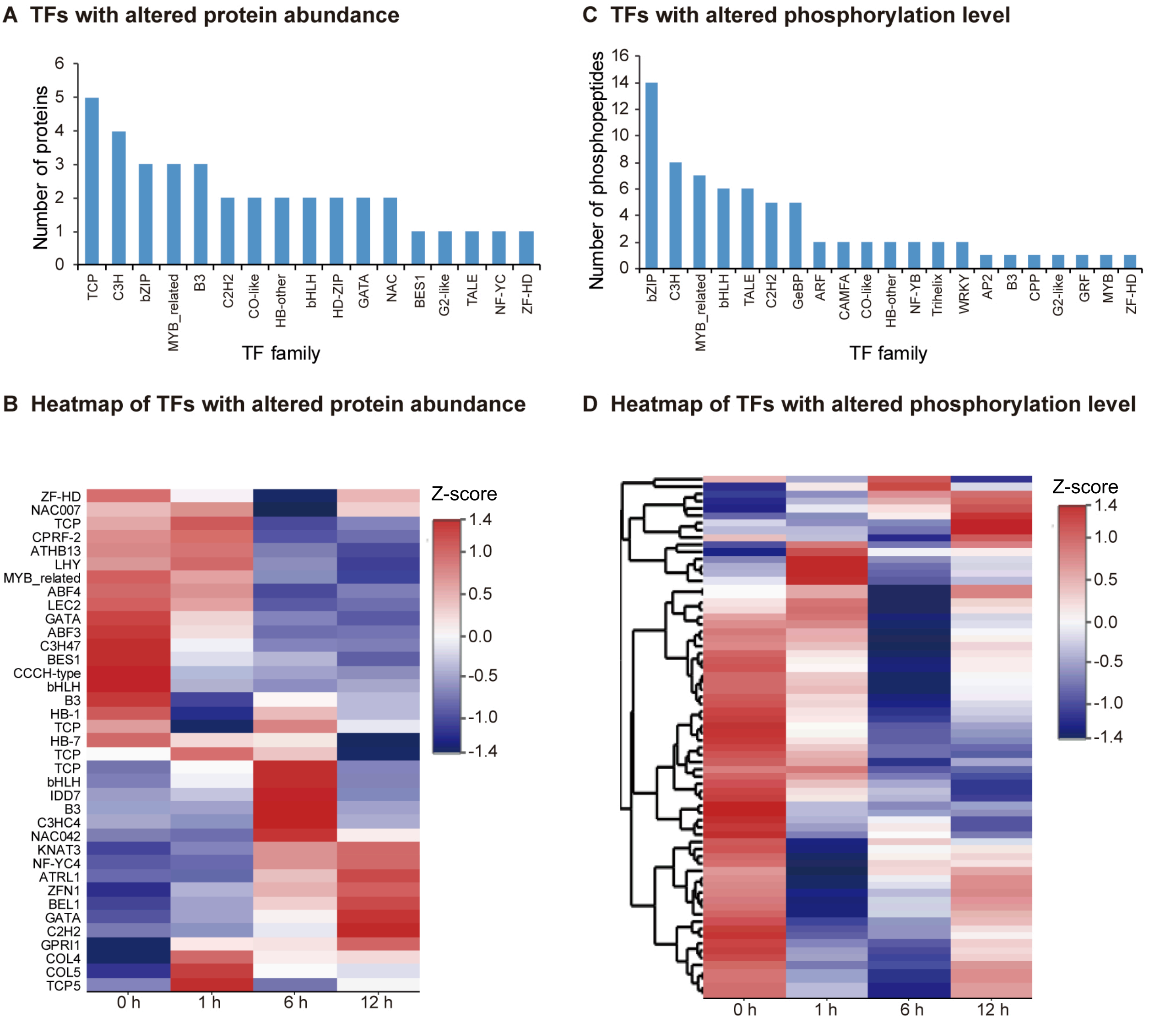
A TFs with altered protein abundance
Number of proteins
TF family
TCP
C3H
bZIP
MYB_related
B3
C2H2
CO-like
HB-other
bHLH
HD-ZIP
GATA
NAC
BES1
G2-like
TALE
NF-YC
ZF-HD
C TFs with altered phosphorylation level
Number of phosphopeptides
TF family
bZIP
C3H
MYB_related
bHLH
TALE
C2H2
GeBP
ARF
CAMFA
CO-like
HB-other
NF-YB
Trihelix
WRKY
AP2
B3
CPP
G2-like
GRF
MYB
ZF-HD
B Heatmap of TFs with altered protein abundance
ZF-HD
NAC007
TCP
CPRF-2
ATHB13
LHY
MYB_related
ABF4
LEC2
GATA
ABF3
C3H47
BES1
CCCH-type
bHLH
B3
HB-1
TCP
HB-7
TCP
TCP
bHLH
IDD7
B3
C3HC4
NAC042
KNAT3
NF-YC4
ATRL1
ZFN1
BEL1
GATA
C2H2
GPRI1
COL4
COL5
TCP5
0 h
1 h
6 h
12 h
Z-score
1.4
1.0
0.5
0.0
-0.5
-1.0
-1.4
D Heatmap of TFs with altered phosphorylation level
0 h
1 h
6 h
12 h
Z-score
1.4
1.0
0.5
0.0
-0.5
-1.0
-1.4
