# Supplementary material for: Large-scale Proteomic and Phosphoproteomic Analyses of Maize Seedling Leaves During De-etiolation
Source: Genomics Proteomics Bioinformatics. 2020 Dec 30;18(4):397–414. doi: 10.1016/j.gpb.2020.12.004 (PMC8242269; doi:10.1016/j.gpb.2020.12.004)
Supplement: Supplementary Figure S5 — Alignment of the AtPHYB and ZmPHYB proteins. The amino acid sequences encoded by AtPHYB (AT2G18790.1) and two ZmPHYBs (GRMZM2G092174_P01 and GRMZM2G124532_P03) were aligned. The “S” residues outlined in red boxes are the phosphorylation sites Ser86 identified in Arabidopsis and Ser76 identified in Zea mays. [file mmc5.pdf]

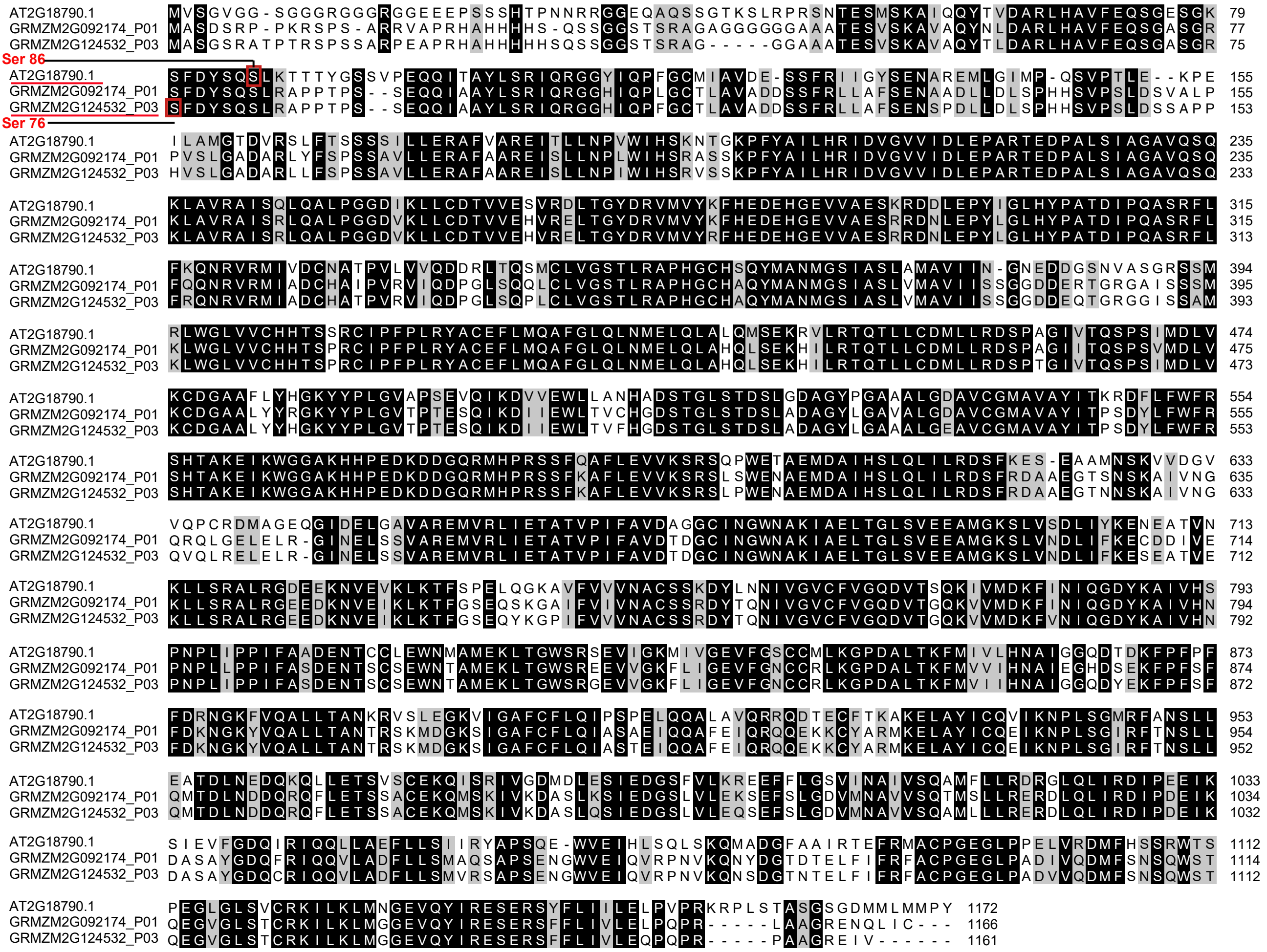
AT2G18790.1 MVSGVGG-SGGGRGGGRGGEEEPSSSHTPNNRRGGEQAQSSGTKSLRPRSNTESMSKAIQQYTVDARLHAVFEQSGESGK 79
GRMZM2G092174_P01 MASDSRP-PKRSPS-ARRVAPRHAHHHHS-QSSGGSTSRAGAGGGGGAAATESVSKAVAQYNLDARLHAVFEQSGASGR 77
GRMZM2G124532_P03 MASGSRATPTRSPSSARPEAPRHAHHHHHSQSSGGSTSRAG-----GGAAATESVSKAVAQYTLDARLHAVFEQSGASGR 75
Ser 86
AT2G18790.1 SFDYSQSLKTTTYGSSVPEQQITAYLSRIQRGGYIQPFGCMIAVDE-SSFRIIGYSENAREMLGIMP-QSVPTLE--KPE 155
GRMZM2G092174_P01 SFDYSQSLRAPPTPS--SEQQIAAYLSRIQRGGHIQPLGCTLAVADDSSFRLLAFSENAADLLDLSPHHSVPSLDSVALP 155
GRMZM2G124532_P03 SFDYSQSLRAPPTPS--SEQQIAAYLSRIQRGGHIQPFGCTLAVADDSSFRLLAFSENSPDLLDLSPHHSVPSLDSSAPP 153
Ser 76
AT2G18790.1 ILAMGTDVRSLFTSSSSILLERAFVAREITLLNPVWIHSKNTGKPFYAILHRIDVGVVIDLEPARTEDPALSIAGAVQSQ 235
GRMZM2G092174_P01 PVSLGADARLYFSPSSAVLLERAFAAREISLLNPLWIHSRASSKPFYAILHRIDVGVVIDLEPARTEDPALSIAGAVQSQ 235
GRMZM2G124532_P03 HVSLGADARLLFSPSSAVLLERAFAAREISLLNPIWIHSRVSSKPFYAILHRIDVGVVIDLEPARTEDPALSIAGAVQSQ 233
AT2G18790.1 KLAVRAISQLQALPGGDIKLLCDTVVESVRDLTGYDRVMVYKFHEDEHGEVVAESKRDDLEPYIGLHYPATDIPQASRFL 315
GRMZM2G092174_P01 KLAVRAISRLQALPGGDVKLLCDTVVEHVRELTGYDRVMVYKFHEDEHGEVVAESRRDNLEPYLGLHYPATDIPQASRFL 315
GRMZM2G124532_P03 KLAVRAISRLQALPGGDVKLLCDTVVEHVRELTGYDRVMVYRFHEDEHGEVVAESRRDNLEPYLGLHYPATDIPQASRFL 313
AT2G18790.1 FKQNRVRMIVDCNATPVLVVQDDRLTQSMCLVGSTLRAPHGCHSQYMANMGSIASLAMAVIIN-GNEDDGSNVASGRSSM 394
GRMZM2G092174_P01 FQQNRVRMIADCHAIPVRVIQDPGLSQQLCLVGSTLRAPHGCHAQYMANMGSIASLVMAVIISSGGDDERTGRGAISSSM 395
GRMZM2G124532_P03 FRQNRVRMIADCHATPVRVIQDPGLSQPLCLVGSTLRAPHGCHAQYMANMGSIASLVMAVIISSGGDDEQTGRGGISSAM 393
AT2G18790.1 RLWGLVVCHHTSSRCIPFPLRYACEFLMQAFGLQLNMELQLALQMSEKRVLRTQTLLCDMLLRDSPAGIVTQSPSIMDLV 474
GRMZM2G092174_P01 KLWGLVVCHHTSPRCIPFPLRYACEFLMQAFGLQLNMELQLAHQLSEKHILRTQTLLCDMLLRDSPAGIITQSPSVMDLV 475
GRMZM2G124532_P03 KLWGLVVCHHTSPRCIPFPLRYACEFLMQAFGLQLNMELQLAHQLSEKHILRTQTLLCDMLLRDSPTGIVTQSPSIMDLV 473
AT2G18790.1 KCDGAAFLYHGKYYPLGVAPSEVQIKDVVEWLLANHADSTGLSTDSLGDAGYPGAAALGDAVCGMAVAYITKRDFLFWFR 554
GRMZM2G092174_P01 KCDGAALYYRGKYYPLGVTPTESQIKDIIEWLTVCHGDSTGLSTDSLADAGYLGAVALGDAVCGMAVAYITPSDYLFWFR 555
GRMZM2G124532_P03 KCDGAALYYHGKYYPLGVTPTESQIKDIIEWLTVFHGDSTGLSTDSLADAGYLGAAALGEAVCGMAVAYITPSDYLFWFR 553
AT2G18790.1 SHTAKEIKWGGAKHHPEDKDDGQRMHPRSSFQAFLEVVKSRSQPWETAEMDAIHSLQLILRDSFKES-EAAMNSKVVDGV 633
GRMZM2G092174_P01 SHTAKEIKWGGAKHHPEDKDDGQRMHPRSSFKAFLEVVKSRSLSWENAEMDAIHSLQLILRDSFRDAAEGTSNSKAIVNG 635
GRMZM2G124532_P03 SHTAKEIKWGGAKHHPEDKDDGQRMHPRSSFKAFLEVVKSRSLPWENAEMDAIHSLQLILRDSFRDAAEGTNNSKAIVNG 633
AT2G18790.1 VQPCRDMAGEQGIDELGAVAREMVRLIETATVPIFAVDAGGCINGWNAKIAELTGLSVEEAMGKSLVSDLIYKENEATVN 713
GRMZM2G092174_P01 QRQLGELELR-GINELSSVAREMVRLIETATVPIFAVDTDGCINGWNAKIAELTGLSVEEAMGKSLVNDLIFKECDDIVE 714
GRMZM2G124532_P03 QVQLRELELR-GINELSSVAREMVRLIETATVPIFAVDTDGCINGWNAKIAELTGLSVEEAMGKSLVNDLIFKESEATVE 712
AT2G18790.1 KLLSRALRGDEEKNVEVKLKTFSPELQGKAVFVVVNACSSKDYLNNIVGVCFVGQDVTSQKIVMDKFINIQGDYKAIVHS 793
GRMZM2G092174_P01 KLLSRALRGEEDKNVEIKLKTFGSEQSKGAIFVIVNACSSRDYTQNIVGVCFVGQDVTGQKVVMDKFINIQGDYKAIVHN 794
GRMZM2G124532_P03 KLLSRALRGEEDKNVEIKLKTFGSEQYKGPIFVVVNACSSRDYTQNIVGVCFVGQDVTGQKVVMDKFVNIQGDYKAIVHN 792
AT2G18790.1 PNPLIPPIFAADENTCCLEWNMAMEKLTGWSRSEVIGKMIVGEVFGSCCMLKGPDALTKFMIVLHNAIGGQDTDKFPFPF 873
GRMZM2G092174_P01 PNPLLPPIFASDENTSCSEWNTAMEKLTGWSREEVVGKFLIGEVFGNCCRLKGPDALTKFMVVIHNAIEGHDSEKFPFSF 874
GRMZM2G124532_P03 PNPLIPPIFASDENTSCSEWNTAMEKLTGWSRGEVVGKFLIGEVFGNCCRLKGPDALTKFMVIIHNAIGGQDYEKFPFSF 872
AT2G18790.1 FDRNGKFVQALLTANKRVSLEGKVIGAFCFLQIPSPELQQALAVQRRQDTECFTKAKELAYICQVIKNPLSGMRFANSLL 953
GRMZM2G092174_P01 FDKNGKYVQALLTANTRSKMDGKSIGAFCFLQIASAEIQQAFEIQRQQEKKCYARMKELAYICQEIKNPLSGIRFTNSLL 954
GRMZM2G124532_P03 FDKNGKYVQALLTANTRSKMDGKSIGAFCFLQIASTEIQQAFEIQRQQEKKCYARMKELAYICQEIKNPLSGIRFTNSLL 952
AT2G18790.1 EATDLNEDQKQLLETSVSCEKQISRIVGDMDLESIEDGSFVLKREEFFLGSVINAIVSQAMFLLRDRGLQLIRDIPEEIK 1033
GRMZM2G092174_P01 QMTDLNDDQRQFLETSSACEKQMSKIVKDASLKSIEDGSLVLEKSEFSLGDVMNAVVSQTMSLLRERDLQLIRDIPDEIK 1034
GRMZM2G124532_P03 QMTDLNDDQRQFLETSSACEKQMSKIVKDASLQSIEDGSLVLEQSEFSLGDVMNAVVSQAMLLLRERDLQLIRDIPDEIK 1032
AT2G18790.1 SIEVFGDQIRIQQLLAEFLLSIIRYAPSQE-WVEIHLSQLSKQMADGFAAIRTEFRMACPGEGLPPELVRDMFHSSRWTS 1112
GRMZM2G092174_P01 DASAYGDQFRIQQVLADFLLSMAQSAPSENGWVEIQVRPNVKQNYDGTDTELFIFRFACPGEGLPADIVQDMFSNSQWST 1114
GRMZM2G124532_P03 DASAYGDQCRIQQVLADFLLSMVRSAPSENGWVEIQVRPNVKQNSDGTNTELFIFRFACPGEGLPADVVQDMFSNSQWST 1112
AT2G18790.1 PEGLGLSVCRKILKLMNGEVQYIRESERSYFLIILELPVPRKRPLSTASGSGDMMLMMPY 1172
GRMZM2G092174_P01 QEGVGLSTCRKILKLMGGEVQYIRESERSFFLIVLELPQPR-----LAAGRENQLIC--- 1166
GRMZM2G124532_P03 QEGVGLSTCRKILKLMGGEVQYIRESERSFFLIVLEQPQPR-----PAAGREIV------ 1161
