# Supplementary material for: Large-scale Proteomic and Phosphoproteomic Analyses of Maize Seedling Leaves During De-etiolation
Source: Genomics Proteomics Bioinformatics. 2020 Dec 30;18(4):397–414. doi: 10.1016/j.gpb.2020.12.004 (PMC8242269; doi:10.1016/j.gpb.2020.12.004)
Supplement: Supplementary Figure S6 — Alignment of the AtCRY2 and ZmCRY2 proteins. The amino acid sequences encoded by AtCRY2 (AT1G04400.2) and two ZmCRY2 transcripts (GRMZM2G172152_T01 and GRMZM2G172152_T02) were aligned. The “S” residues outlined in red boxes are the phosphorylation sites identified in Arabidopsis and Zea mays. The red asterisks indicate the conserved phosphorylation sites. [file mmc6.pdf]

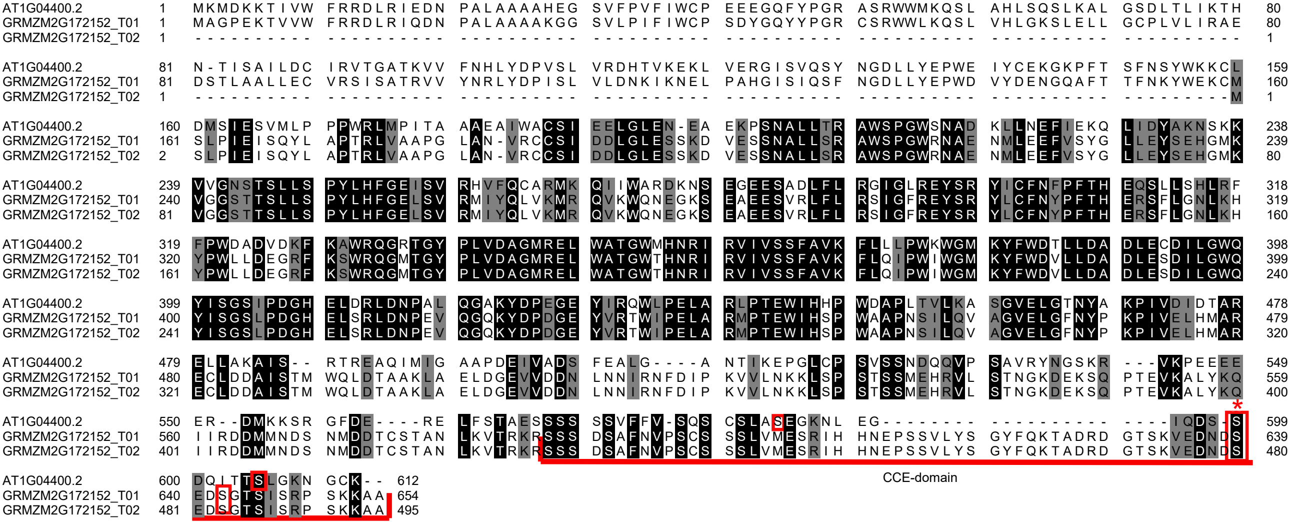
AT1G04400.2 1 MKMDKKTIVW FRRDLRIEDN PALAAAAHEG SVFPVFIWCP EEEGQFYPGR ASRWWMKQSL AHLSQSLKAL GSDLTLIKTH 80
GRMZM2G172152_T01 1 MAGPEKTVVW FRRDLRIQDN PALAAAAKGG SVLPIFIWCP SDYGQYYPGR CSRWWLKQSL VHLGKSLELL GCPLVLIRAE 80
GRMZM2G172152_T02 1 ---------- ---------- ---------- ---------- ---------- ---------- ---------- ---------- 1
AT1G04400.2 81 N-TISAILDC IRVTGATKVV FNHLYDPVSL VRDHTVKEKL VERGISVQSY NGDLLYEPWE IYCEKGKPFT SFNSYWKKCL 159
GRMZM2G172152_T01 81 DSTLAALLEC VRSISATRVV YNRLYDPISL VLDNKIKNEL PAHGISIQSF NGDLLYEPWD VYDENGQAFT TFNKYWEKCM 160
GRMZM2G172152_T02 1 ---------- ---------- ---------- ---------- ---------- ---------- ---------- ---------M 1
AT1G04400.2 160 DMSIESVMLP PPWRLMPITA AAEAIWACSI EELGLEN-EA EKPSNALLTR AWSPGWSNAD KLLNEFIEKQ LIDYAKNSKK 238
GRMZM2G172152_T01 161 SLPIEISQYL APTRLVAAPG LAN-VRCCSI DDLGLESSKD VESSNALLSR AWSPGWRNAE NMLEEFVSYG LLEYSEHGMK 239
GRMZM2G172152_T02 2 SLPIEISQYL APTRLVAAPG LAN-VRCCSI DDLGLESSKD VESSNALLSR AWSPGWRNAE NMLEEFVSYG LLEYSEHGMK 80
AT1G04400.2 239 VVGNSTSLLS PYLHFGEISV RHVFQCARMK QIIWARDKNS EGEESADLFL RGIGLREYSR YICFNFPFTH EQSLLSHLRF 318
GRMZM2G172152_T01 240 VGGSTTSLLS PYLHFGELSV RMIYQLVKMR QVKWQNEGKS EAEESVRLFL RSIGFREYSR YLCFNYPFTH ERSFLGNLKH 319
GRMZM2G172152_T02 81 VGGSTTSLLS PYLHFGELSV RMIYQLVKMR QVKWQNEGKS EAEESVRLFL RSIGFREYSR YLCFNYPFTH ERSFLGNLKH 160
AT1G04400.2 319 FPWDADVDKF KAWRQGRTGY PLVDAGMREL WATGWMHNRI RVIVSSFAVK FLLLPWKWGM KYFWDTLLDA DLECDILGWQ 398
GRMZM2G172152_T01 320 YPWLLDEGRF KSWRQGMTGY PLVDAGMREL WATGWTHNRI RVIVSSFAVK FLQIPWIWGM KYFWDVLLDA DLESDILGWQ 399
GRMZM2G172152_T02 161 YPWLLDEGRF KSWRQGMTGY PLVDAGMREL WATGWTHNRI RVIVSSFAVK FLQIPWIWGM KYFWDVLLDA DLESDILGWQ 240
AT1G04400.2 399 YISGSIPDGH ELDRLDNPAL QGAKYDPEGE YIRQWLPELA RLPTEWIHHP WDAPLTVLKA SGVELGTNYA KPIVDIDTAR 478
GRMZM2G172152_T01 400 YISGSLPDGH ELSRLDNPEV QGQKYDPDGE YVRTWIPELA RMPTEWIHSP WAAPNSILQV AGVELGFNYP KPIVELHMAR 479
GRMZM2G172152_T02 241 YISGSLPDGH ELSRLDNPEV QGQKYDPDGE YVRTWIPELA RMPTEWIHSP WAAPNSILQV AGVELGFNYP KPIVELHMAR 320
AT1G04400.2 479 ELLAKAIS-- RTREAQIMIG AAPDEIVADS FEALG----A NTIKEPGLCP SVSSNDQQVP SAVRYNGSKR ---VKPEEEE 549
GRMZM2G172152_T01 480 ECLDDAISTM WQLDTAAKLA ELDGEVVDDN LNNIRNFDIP KVVLNKKLSP STSSMEHRVL STNGKDEKSQ PTEVKALYKQ 559
GRMZM2G172152_T02 321 ECLDDAISTM WQLDTAAKLA ELDGEVVDDN LNNIRNFDIP KVVLNKKLSP STSSMEHRVL STNGKDEKSQ PTEVKALYKQ 400
*
AT1G04400.2 550 ER--DMKKSR GFDE----RE LFSTAESSSS SSVFFV-SQS CSLASEGKNL EG-------- ---------- ----IQDS-S 599
GRMZM2G172152_T01 560 IIRDDMMNDS NMDDTCSTAN LKVTRKRSSS DSAFNVPSCS SSLVMESRIH HNEPSSVLYS GYFQKTADRD GTSKVEDNDS 639
GRMZM2G172152_T02 401 IIRDDMMNDS NMDDTCSTAN LKVTRKRSSS DSAFNVPSCS SSLVMESRIH HNEPSSVLYS GYFQKTADRD GTSKVEDNDS 480
AT1G04400.2 600 DQLTTSLGKN GCK-- 612
GRMZM2G172152_T01 640 EDSGTSISRP SKKAA 654
GRMZM2G172152_T02 481 EDSGTSISRP SKKAA 495
CCE-domain
