# Supplementary material for: Large-scale Proteomic and Phosphoproteomic Analyses of Maize Seedling Leaves During De-etiolation
Source: Genomics Proteomics Bioinformatics. 2020 Dec 30;18(4):397–414. doi: 10.1016/j.gpb.2020.12.004 (PMC8242269; doi:10.1016/j.gpb.2020.12.004)
Supplement: Supplementary Figure S7 — Alignment of the AtCaS and ZmCaS proteins. The amino acid sequences of AtCaS (AT5G23060.1) and ZmCaS (GRMZM2G122715_T02) were aligned. The “T” outlined by a red box is the conserved phosphorylation site identified in both the Arabidopsis and Zea mays proteins. [file mmc7.pdf]

```
AT5G23060.1          1    MAMAEMATKS SLSAKLTLPS SSTKKTLSLR QVSVSLPTST SISLLSLFAS PPHEAKAAVS IPKDQIVSSL TEVEKTINQV  80
GRMZM2G122715_T02    1    MAPVPVSVSA TLAPPPAAPP KATSRSWERR APADAAFAAA SSVAISAALL TLTPAAPAAA LSKEDVAGSL TKAVDTVSQA  80

AT5G23060.1          81   QETGSSVFDA TQRVFQVVGD ALKPALDTAL PIAKQAGEEA MKLASPAFSE ASKKAQEAMQ SSGFDSEPVF NAAKTVTDVA  160
GRMZM2G122715_T02    81   IDVGGKAAEQ VAAVLKALGE AVKP----AL PVLKSASDEA LKLAAPVVSA ASKQATEALQ GAGVDPAPVL SVAKTA---A  153

AT5G23060.1          161  QQTSKAIEDA KPIASSTMDT ISSADPSVIV VAAGAAFLAY LLLPPVFSAI SFNFRGYKGD LTPAQTLDLL CTKNYLMVDI  240
GRMZM2G122715_T02    154  EQSTKVIDAA KPVASAAVET ITSLGPEDYV VAAGAAFLAY LLVPPVWSLV SSSLRGYKGD LTPAQALDKV TTQGYVLIDV  233

AT5G23060.1          241  RSEKDKEKAG IPRLPSNAKN RVISIPLEEL PNKVKGIVRN SKRVEAEIAA LKISYLKKIN KGSNIIILDS YTDSAKIVAK  320
GRMZM2G122715_T02    234  RSDKDKAKAG LPQLPSNAKN KLVSVPLEDL PSKLKGMVRN AKKAEAEIAA LKISYLKKIG KGSNVIIMDS YSDVAKTVAK  313

AT5G23060.1          321  TLKVLGYKNC YIVTDGFSGG RGWLQSRLGT DSYNFSFAQV LSPSRIIPAA S-RSFGTRSG ---------- ---TKFLPS-  385
GRMZM2G122715_T02    314  TLDSVGFKNC WVMAGGFSGR KGWAQSRLGT DSYNLSVVEV VTPSRVIPAV AGRRTGTTAA RIGTASSASR ATTRKLLPGG  393

AT5G23060.1          386  SD  387
GRMZM2G122715_T02    394  VD  395
```
